# Combining multiple imputation and meta-analysis with individual participant data

**DOI:** 10.1002/sim.5844

**Published:** 2013-05-24

**Authors:** Stephen Burgess, Ian R White, Matthieu Resche-Rigon, Angela M Wood

**Affiliations:** Department of Public Health & Primary Care, Strangeways Research Laboratory2 Worts Causeway, Cambridge, CB1 8RN, U.K.

**Keywords:** missing data, multiple imputation, meta-analysis, individual participant data, Rubin's rules

## Abstract

Multiple imputation is a strategy for the analysis of incomplete data such that the impact of the missingness on the power and bias of estimates is mitigated. When data from multiple studies are collated, we can propose both within-study and multilevel imputation models to impute missing data on covariates. It is not clear how to choose between imputation models or how to combine imputation and inverse-variance weighted meta-analysis methods. This is especially important as often different studies measure data on different variables, meaning that we may need to impute data on a variable which is systematically missing in a particular study. In this paper, we consider a simulation analysis of sporadically missing data in a single covariate with a linear analysis model and discuss how the results would be applicable to the case of systematically missing data. We find in this context that ensuring the congeniality of the imputation and analysis models is important to give correct standard errors and confidence intervals. For example, if the analysis model allows between-study heterogeneity of a parameter, then we should incorporate this heterogeneity into the imputation model to maintain the congeniality of the two models. In an inverse-variance weighted meta-analysis, we should impute missing data and apply Rubin's rules at the study level prior to meta-analysis, rather than meta-analyzing each of the multiple imputations and then combining the meta-analysis estimates using Rubin's rules. We illustrate the results using data from the Emerging Risk Factors Collaboration.

## 1. Introduction

Missing data are data whose values are not available. This may be for a number of different reasons both within and outside the control of investigators. Factors within their control tend to lead to systematic patterns of missing data, such as data on a variable being missing for an entire subset of the study population. This may be because the measurement of a variable was only undertaken in a few studies, for example, due to the cost of measurement. Factors outside the control of the investigators tend to lead to sporadically missing data, where data on a variable are missing for a few individuals with no clear pattern to the missingness.

Missing data are classified as missing completely at random (MCAR), missing at random (MAR), or missing not at random (MNAR) depending on whether the probability of data being missing is independent of the true values of the missing data (MCAR), depends only on observed data (MAR), or depends additionally on unobserved data (MNAR) [1,2]. If the data are MCAR, then the missing values are distributed identically to the measured values. If the data are MAR, then the distributions of the missing and measured values are the same conditional on measured covariates. If the data are MNAR, the conditional distribution of the missing values differs from that of the measured values.

There are several possible approaches with missing data. The most common approach is to ignore individuals with missing data entirely: a complete-case analysis. More sophisticated approaches are available, such as multiple imputation [3]. Multiple imputation under a MAR assumption is increasingly being used in applied research because of recent software development. In multiple imputation, missing values are imputed several times by drawing random values from the conditional distribution of the missing values according to a specified imputation model using observed data values to form a completed dataset. The parameter estimates and standard errors from each of these imputed datasets are combined using formulae known as Rubin's rules [4]. There are two main advantages to a multiple imputation analysis over a complete-case analysis:

1. *Power*: Observations with partial missingness may still be informative for the estimate of interest, especially if missingness is in a single variable. An efficient analysis should include all relevant information.
2. *Bias*: If the missing data are MAR, a complete-case analysis can introduce bias, whereas correctly specified multiple imputation estimates are unbiased [5].

In this paper, we consider the specific context of multiple imputation for missing data in an individual participant data (IPD) meta-analysis [6]. A meta-analysis is an analysis of data from multiple sources to give a single pooled value representing the overall estimate of the parameter of interest using the totality of the available data. Often, by necessity, a meta-analysis is performed on summarized data published by each study. In an IPD meta-analysis, the original data on the study participants is available for analysis. This facilitates hierarchical analyses, where the analysis of multilevel data can be performed in a single step [7], as opposed to the common two-stage inverse-weighted meta-analysis method.

The main difficulty with performing and interpreting meta-analyses is between-study variability [8]. This consists of both statistical heterogeneity due to differences in populations, such that coefficients cannot realistically be assumed to be constant across studies, and variability due to the investigators, such as the choice of variables measured in each study or the definition of variables. IPD enable the assessment of statistical heterogeneity and the standardization of analyses across studies [9]. Additionally, detailed analyses can be performed with individual-level data, which would not generally be possible with summarized data, such as multiple imputation [10,11].

The imputation of missing data presents specific challenges in a meta-analysis context. For example, if a covariate represents an important confounder for an association, missing data methods can be used to impute sporadically missing data in the covariate, although it is not clear whether it would be optimal to impute data in each study separately or in all studies using a hierarchical model. If the covariate has not been measured in a study, it is unclear how to impute data on this variable using information from other studies. Previous work has shown that imputation of covariates across multiple studies can lead to inconsistencies in estimation [12].

The structure of this paper is as follows. We first introduce methods for the analysis of data from multiple studies and the imputation of missing data in this context (Section 2). Two particular issues considered are the following: (i) the congeniality of the imputation and analysis models [13] and (ii) the correct order to apply the combination of imputation estimates using Rubin's rules and the pooling of study estimates using an inverse-variance weighted meta-analysis. We present a simulation study to investigate the behavior of estimates using the analysis and imputation methods previously introduced (Section 3). We illustrate the methods with an analysis of the association of low-density lipoprotein cholesterol (LDL-C) with blood pressure using data from the Emerging Risk Factors Collaboration (ERFC) [14] (Section 4). We conclude by discussing the findings of the paper and potential avenues for future work (Section 5). The methods and simulations considered in this paper mainly relate to sporadically missing data. Issues relating to systematically missing data are left for discussion.

## 2. Meta-analysis and multiple imputation models

We consider a situation where we have two continuous covariates (*X*_1_ and *X*_2_) and a continuous outcome (*Y*) in multiple studies. We present methods for the analysis of such data and for the imputation of missing data on one of the covariates.

### 2.1. Meta-analysis models

The three meta-analysis methods considered here are a homoscedastic stratified analysis, an inverse-variance weighted fixed-effect analysis, and an inverse-variance weighted random-effects analysis using the DerSimonian and Laird estimate of between-study heterogeneity [15].

In the homoscedastic stratified analysis, we estimate the following model using linear regression:


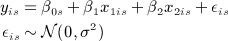
(1)

where the subscript *i* is used to index individual participants and *s* to index studies. This model assumes constant coefficients *β*_1_ and *β*_2_ in the regression model for both *X*_1_ and *X*_2_, with fixed study-specific intercepts *β*_0*s*_. We could perform a more sophisticated heteroscedastic stratified analysis using a hierarchical model to allow for heterogeneity in the variance of the error between studies (that is, 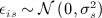) or using random study-level effects on the regression coefficients *β*_1_ and *β*_2_. We do not consider these analyses here for reasons of focus and brevity of presentation.

In the inverse-variance weighted analyses, we initially fit a separate model in each study using linear regression:


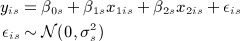
(2)

We then combine the estimated coefficients 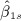 and 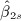 using inverse-variance weighting. We can use either a fixed-effect or a random-effects model [16]. In this paper, we consider that an estimate either *β*_1_ or *β*_2_ is of interest; we do not consider multivariate meta-analyses for the joint distribution of the estimates of *β*_1_ and *β*_2_ [17].

### 2.2. Multiple-imputation models

We investigate two models for imputing missing data: a stratified model and a within-study model.

In the (homoscedastic) stratified imputation method, we impute missing data by using the following model with fixed study-level intercepts:


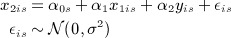
(3)

This model uses the same *α*_1_ and *α*_2_ parameters in each study, fixed study-level intercept terms, and assumes that the error distribution is homogeneous across studies. Aside from its simplicity and availability in a wide range of statistical software packages, an advantage of this model is the ability to impute data on a systematically missing covariate. In this case, we cannot estimate the study-specific intercept (*α*_0*s*_) from data. For the linear analysis models considered in this paper, we can fix *α*_0*s*_ at an arbitrary value (say, zero) as the value of *α*_0*s*_ affects only the study-specific intercept term (*β*_0*s*_) in the analysis model.

In the within-study imputation method, we impute missing data by using the following model:


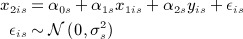
(4)

This model uses different *α*_1*s*_ and *α*_2*s*_ parameters in each study and allows the residual error variance to differ between studies. In practice, we impute data in each study separately.

Alternatively, we could assume a heteroscedastic stratified imputation model, where the same parameters (*α*_1_ and *α*_2_) are used in each study as in equation (3) but the variance of the error distribution 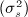 is different in each study. Although this may be a better model in many cases, it is unclear what value of the variance should be taken if no measurements have been made of the covariate in a particular study, although a hierarchical model for the variances may be possible [11]. We did not consider this in this paper because of similarity to the other models considered; additionally, we prioritize models, which are available for implementation in standard software for multiple imputation, for investigation.

### 2.3. Congeniality of imputation and analysis models

An important consideration for a multiple imputation analysis is the congeniality of the models used in imputation and analysis of data. Imputation and analysis models are compatible if a joint model exists under which both models are conditionals [18]. The concept of congeniality, introduced by Meng [13] in the context of multiple imputation, states that as follows: (i) given complete data, the analysis model asymptotically gives the same mean and variance estimates as the posterior mean and variance from a Bayesian joint model, and (ii) given incomplete data, the imputation model gives the same posterior predictive distribution for missing values as the Bayesian joint model. Congeniality is similar to compatibility in a non-Bayesian context with the regularity condition that the priors in the Bayesian model are nonzero over the entire parameter space.

### 2.4. Combining Rubin's rules and inverse-variance weighted meta-analysis

**Figure 1:**
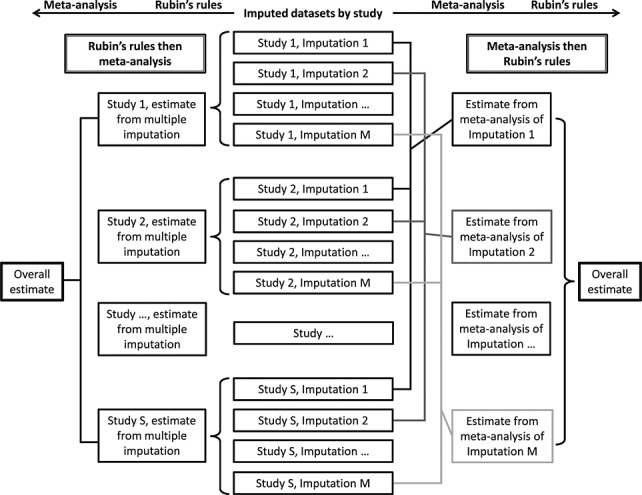
Schematic diagram illustrating the two approaches for combining Rubin's rules and inverse-variance weighted meta-analysis. Curved braces indicate application of Rubin's rules, square braces indicate application of meta-analysis.

In an inverse-variance weighted meta-analysis, there are two ways of producing a single estimate from several multiply imputed studies: pooling across studies by meta-analysis separately for each imputed dataset and then combining the meta-analysis estimates using Rubin's rules, or combining estimates using Rubin's rules for each study and then meta-analyzing the combined estimates across studies. Figure 1 shows a schematic diagram of the two approaches.

## 3. Simulation study

To assess the performance of methods for imputing missing data in a particular meta-analysis context, especially with regard to the issues of congeniality and the order of applying Rubin's rules and meta-analysis in a inverse-variance weighted meta-analysis, we perform a simulation study.

### 3.1. Simulation of data

We simulate data on 30 studies from the following data-generating model:


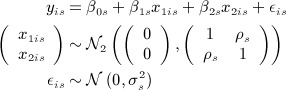
(5)

In the first set of simulations, each study is of equal size with 200 participants in each study. In the second set of simulations, studies are of unequal size with the number of participants in each study ranging from 125 to 275 in steps of five individuals, such that the total number of individuals in both simulations is the same. Supporting Information ‡ presents results from the second simulation with studies of unequal size.

We consider five scenarios, representing different degrees of between-study heterogeneity:

**Table:** 

	Scenario 1	Scenario 2	Scenario 3	Scenario 4	Scenario 5
*β*_0*s*_	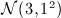	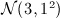	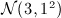	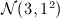	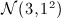
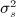	1	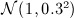	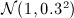	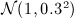	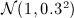
*ρ*_*s*_	0.2	0.2	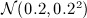	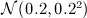	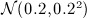
*β*_2*s*_	− 0.6	− 0.6	− 0.6	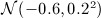	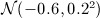
*β*_1*s*_	0.3	0.3	0.3	0.3	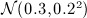

where a numerical value indicates that the parameter took that value for all studies, and a normal distribution 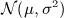 indicates that the values of the parameter in each study were drawn independently from a normal distribution.

Scenario 1 is the most homogeneous model considered, and we add heterogeneity sequentially by drawing the model parameters from a normal distribution to allow for between-study variability. We note that the homoscedastic stratified model given in equation (1) is a correctly specified analysis model in Scenario 1. A heteroscedastic stratified model is a correctly specified analysis model in Scenario 2 (and should give consistent estimates in Scenario 1). The fixed-effect method is correctly specified for *β*_1_ in Scenarios 3 and 4 (and should give consistent estimates for both *β*_1_ and *β*_2_ in Scenarios 1 and 2). We correctly specify the random-effects method for both *β*_1_ and *β*_2_ in Scenario 5 (and should give consistent estimates for both parameters in each of the other scenarios).

Similarly, the stratified imputation model is a correctly specified imputation model in Scenario 1, but not in any of the other scenarios. The within-study imputation model should give consistent imputed values in all scenarios.

In each scenario, we create 1000 simulated datasets for analysis. We assume that *Y* and *X*_1_ have no missing observations and only consider missingness in *X*_2_. If *π*_*is*_ is the probability that the observation *x*_*is*_ is missing, we generate approximately 50% sporadically missing data in *X*_2_ using a MAR model, where missingness depends on the observed value of *X*_1_ (which is correlated with *X*_2_):


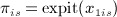
(6)

where expit(*x*) = (1 + exp( − *x*))^ − 1^ is the inverse of the logit function. We use this large missingness rate to illustrate the issues in parameter estimation with missing data more clearly.

In this paper, we used five imputations for each dataset for computational reasons. In a practical application, more imputations would ideally be used. We generated multiply imputed datasets in stata (StataCorp, College Station, Texas, USA) [19]; we performed subsequent analyses of the datasets in r (R Foundation for Statistical Computing, Vienna, Austria) [20].

Tables I and II for the equal sized studies and Tables SA1 and SA2 for the unequal sized studies show the results of stratified, fixed-effects, and random-effects meta-analysis methods. Supporting Information shows alternative tables displaying the same results, but grouped by imputation method rather than scenario. In Tables I and II, we show results from scenarios where the analysis model is misspecified with a shaded background, whereas we show results from scenarios where the imputation model is misspecified in italics.

**Table I:** Simulation study comparing complete-data, complete-case, and multiple imputation analyses with two imputation models to estimate *β*_1_ = 0.3 with thirty (30) equal sized studies using three analysis models in five scenarios with increasing heterogeneity: mean and standard deviation (SD) of estimates, mean standard error (SE), and coverage (Cov %) of the 95% confidence interval. In inverse-variance weighted analyses, it is indicated whether Rubin's rules were applied within each study prior to meta-analysis (RR then MA) or meta-analysis of imputed datasets was performed prior to combining estimates using Rubin's rules (MA then RR).

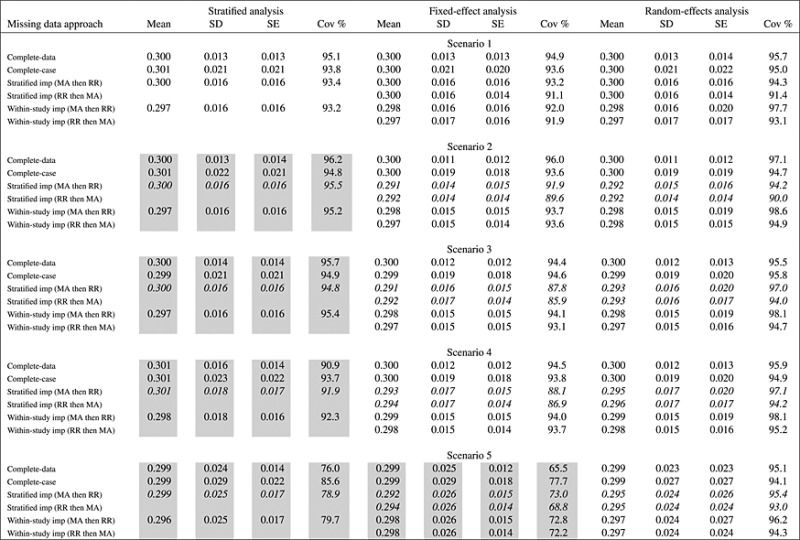

Results from scenarios where the analysis model is misspecified are shown with a shaded background, whereas results from scenarios where the imputation model is misspecified are shown in italics.

**Table II:** Simulation study comparing complete-data, complete-case and multiple imputation analyses with two imputation models to estimate *β*_2_ = − 0.6 with thirty (30) equal sized studies using three analysis models in five scenarios with increasing heterogeneity: mean and standard deviation (SD) of estimates, mean standard error (SE), and coverage (Cov %) of the 95% confidence interval. In inverse-variance weighted analyses, it is indicated whether Rubin's rules were applied within each study prior to meta-analysis (RR then MA) or meta-analysis of imputed datasets was performed prior to combining estimates using Rubin's rules (MA then RR).

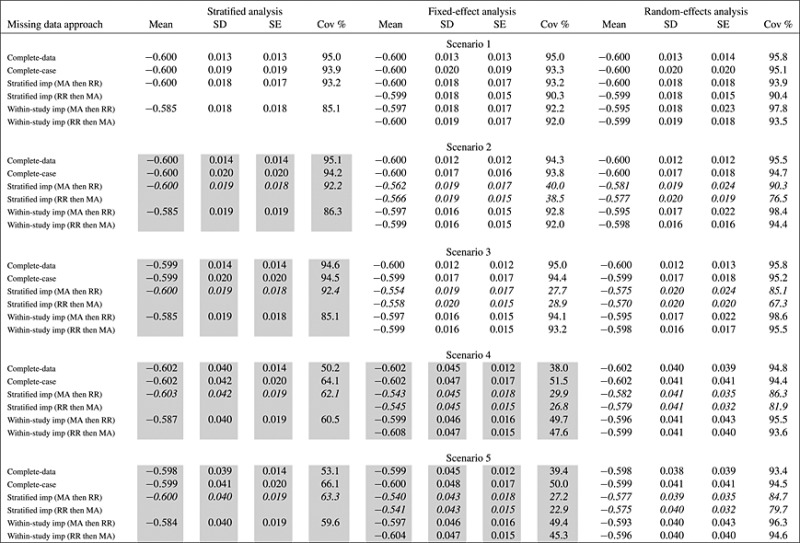

Results from scenarios where the analysis model is misspecified are shown with a shaded background, whereas results from scenarios where the imputation model is misspecified are shown in italics.

### 3.2. Results of complete-data and complete-case analyses

Before considering the imputation of missing data, we discuss complete-data and complete-case analyses estimates of both *β*_1_ (Table I) and *β*_2_ (Table II). In the complete-data analysis, we analyzed data from all individuals prior to the introduction of missing values. In the complete-case analysis, we excluded individuals with missing data values from analysis. We present results for each of the three meta-analysis methods: the mean estimate across simulations, the standard deviation of estimates, the mean of the estimated standard errors, and the coverage of the 95% confidence interval for the true parameter of interest (or the mean of the parameter's distribution when there is heterogeneity in the parameter of interest). In an ideal analysis, the mean estimate across studies should be close to the true parameter value, the empirical standard deviation of the estimates should be close to the mean standard error estimate, and the coverage should be close to 95%. We constructed confidence intervals assuming normal distributions of the parameters of interest.

The pooled estimate from each of the methods shows little bias throughout even when the model is misspecified. The stratified and the fixed-effect analyses give good estimates when the parameter of interest (i.e., *β*_1_ or *β*_2_) is fixed between studies, with some reduction in coverage and less efficient estimates for the stratified method when there is some between-study variability in other parameters. However, both the stratified and the fixed-effect methods underestimate variance when the parameter of interest is heterogeneous. The random-effects meta-analysis method gives marginally larger standard errors than the fixed-effect method when there is no true heterogeneity in the parameter of interest, but gives much better coverage when heterogeneity is present. Coverage in the random-effects meta-analysis is known to be theoretically underestimated because of uncertainty in the heterogeneity not being acknowledged [21]; this does not seem to be a serious issue here as with 30 studies the heterogeneity is well estimated. We note that the loss of information in the complete-case analyses over the complete-data analyses is much less in Scenarios 4 and 5 where there is heterogeneity in the parameter of interest, than it is in the other scenarios.

As seen in these simulations, the complete-case analyses are less efficient than the complete-data analyses. This motivates us to consider methods for the imputation of missing data. We note that analyses, which perform badly in terms of bias or coverage with complete data, are not going to perform well using multiple imputation methods; we should not interpret this as a failure of the multiple imputation method, and we should see correct specification of the analysis model as a first step before choosing between imputation models.

### 3.3. Results of combining Rubin's rules and inverse-variance weighted meta-analysis

To assess the impact of the order of combining Rubin's rules and an inverse-variance weighted meta-analysis, we initially consider estimates with a fixed-effects and a random-effects meta-analysis model using the within-study imputation method, as in this case, the imputation model is correctly specified in all scenarios.

*Within-study imputation, fixed-effect analysis*: With the fixed-effect analyses (ignoring scenarios where a fixed-effect analysis is not appropriate), the coverage is further below the nominal 95% that would be expected by chance when estimates are combined using Rubin's rules and meta-analysis whichever order the processes are undertaken. (The Monte Carlo standard error for the coverage, representing the uncertainty in the simulated results due to the limited number of simulations, is 0.7%.) Additionally, there is a slight but consistent bias toward the null.

The reason that the fixed-effect analyses are undercovered and demonstrate bias is that the imputation of missing data introduces heterogeneity into the estimates of the parameter of interest (say *β*_1_) even when there was no heterogeneity in this parameter in the data-generating model for the studies. Even though the parameter in the data-generating model was the same in all studies, the estimates of the related parameter used in the imputation model for generating imputed data will be different, and so, a fixed-effect analysis model will be misspecified. A study with by chance a larger than average estimate of *β*_1_ in the available data will use this inflated estimate of *β*_1_ (via the related parameter *α*_2_ in the imputation model) to impute the missing data. Hence, the parameter estimate from a study with by chance a larger than average estimate of *β*_1_ will be less precise than from a study with a smaller than average estimate. Pooling the estimates of association results in increased weights for studies with smaller than average estimates of *β*_1_, and a slight downward bias in the combined estimate. This introduction of heterogeneity also leads to slight under-coverage when Rubin's rules are applied before meta-analysis, as the fixed-effect assumption will no longer be valid. The bias and reduction in coverage levels seem to be of similar magnitude when the studies are of equal and unequal size.

*Within-study imputation, random-effects analysis*: With the random-effects analyses, the coverage of the 95% confidence interval when the results are meta-analyzed then Rubin's rules are applied is conservative at 97.7% or greater when there is no heterogeneity in the parameter of interest, with mean standard error consistently larger than the standard deviance of the estimates. When the results are combined using Rubin's rules then meta-analyzed, the coverage is close to the nominal 95% when there is no effect heterogeneity. When there is heterogeneity in the parameter of interest, random-effects meta-analysis is known to give slightly over-narrow confidence intervals as stated in Section 3.2. However, this is an issue with the meta-analysis method, not with the imputation method, and the coverage is close to that achieved in the complete-data analysis. Additionally, there is a slight but consistent bias towards the null.

The reason for the overly conservative coverage is that when the multiple imputations are made, additional heterogeneity is introduced into the imputed datasets. If the imputed datasets are meta-analyzed before combining by Rubin's rules, then the heterogeneity of the meta-analysis results represents the sum of the true heterogeneity between the studies and the heterogeneity introduced due to the imputation process. If the imputed datasets are combined for each study using Rubin's rules before the meta-analysis, then each study estimate after combining reflects the true uncertainty of the estimate using all the data in the study.

*Stratified imputation*: If we consider the stratified imputation model in Scenario 1, the only scenario in which this imputation model is correctly specified, then a congenial analysis requires the meta-analysis to be performed before the application of Rubin's rules. This is because missing data in each study is imputed conditional on data in other studies, inducing a dependence between the imputed data values in different studies, which is not accounted for when Rubin's rules are applied at the study level. The inverse-variance weighted analysis models assume that estimates of the parameter of interest from each study are independent. In this case, the fixed-effect analysis still has slightly low coverage, whereas the random-effects analysis has correct coverage levels.

### 3.4. Results of comparison of imputation models

To assess the impact of different imputation methods on estimates, we compare the performance of estimates from each of the analysis methods.

In general, the efficiency of the multiple imputation analyses for *β*_1_ is greater than that of the complete-case and slightly below than that of the complete-data analyses. The efficiency for *β*_2_ is similar to that of the complete-case analyses, with some slight improvement especially when there is heterogeneity in the parameter. We see that the results obtained are most sensitive to the choice of imputation method.

*Stratified imputation*: The results using the stratified imputation method for the inverse-variance weighted analyses show bias in all scenarios except Scenario 1, where there is little heterogeneity between the studies. This is especially marked for estimates of *β*_2_. The coverage is underestimated, with the mean standard error being generally less than the standard deviation of the estimates, even in Scenario 1. This is because the imputation induces a correlation between data values in different studies, which is not acknowledged in an inverse-variance weighted analysis model. Although the stratified analysis method is misspecified in all scenarios except Scenario 1, the results for this method are not so bad, with minimal bias. This may reflect the congeniality of the imputation and analysis models. The inverse-variance weighted meta-analysis models do not correspond to the imputation model, and so are uncongenial. It seems that the heteroscedasticity introduced from Scenario 2 onwards, which makes the imputation model misspecified, is the key feature of the generating model, which introduces bias into the inverse-variance weighted results.

*Within-study imputation*: Using the within-study imputation method, the stratified analysis method gives biased estimates. The method does not perform as badly in Table I as the inverse-variance weighted methods with the stratified imputation method, although bias is more considerable in Table II. We described the behavior of estimates from inverse-variance weighted methods with the within-study imputation method in Section 3.3. In Scenario 1, using the within-study imputation and the stratified analysis models, the finding that there is bias and that the coverage is low runs contrary to the conventional advice in multiple imputation that the imputation model can be more detailed than the analysis model [22]. In this scenario, the models should both lead to consistent estimates, and the imputation model is larger than the analysis model. However, there is bias, and the coverage is lower than the nominal 95% level.

We conclude that the stratified imputation method should be avoided when there is heterogeneity between studies. Koopman *et al*. and Andridge made a similar finding in an applied study with a logistic analysis model [12] and in the context of a cluster randomized trial [23], respectively. This is unfortunate, as the stratified imputation model provides a method for imputing data on a covariate, which is completely missing in a particular study [24]. In the complete absence of data on a covariate, we must make strong assumptions not only about the relation between the covariate and other variables in the model but also about the error distribution of the covariate. We return to this issue in the discussion.

### 3.5. Results with different numbers of studies

In response to a referee's concern that the results of the simulation study may be different when fewer studies are including in a meta-analysis, we additionally performed the simulation study with the same parameters, except with five studies and with 10 studies. Tables SA3–SA6 shows the results. Similar conclusions are reached in this case: Stratified analysis models are undercovered when there is heterogeneity between studies, even when the parameter of interest is fixed; stratified imputation models for inverse-variance weighted analysis models result in bias and poor coverage properties; within-study imputation models for a fixed-effect analysis model result in poor coverage; within-study imputation models for a random-effects analysis model result in poor coverage when the meta-analysis is performed before combining imputation estimates using Rubin's rules (MA then RR), but coverage is similar to that of the complete-data analysis when the study-specific imputation estimates are combined using Rubin's rules prior to meta-analysis (RR then MA). We underestimate the main difference from the analysis with 30 studies that the coverage of the complete-data analyses using a random-effects analysis model (and therefore the coverage of a correctly specified congenial multiple imputation analysis) when there is heterogeneity in the parameter of interest. As previously stated, this is a known feature of random-effects meta-analysis when there are few studies and the between-study heterogeneity is poorly estimated. This can be mitigated by using a *t*-distribution rather than a normal distribution to form confidence intervals [21].

## 4. Example: the association of low-density lipoprotein cholesterol with blood pressure

We illustrate our findings with data from the ERFC on 53,723 participants from 10 studies for the association of LDL-C (units mmol/L) with systolic blood pressure (units mmHg) using body mass index (units kg/m ^2^) as a covariate. Subjects that were taken are those with complete data on systolic blood pressure and body mass index in studies with data on LDL-C. We introduced missing data on LDL-C for 20% of participants in each study by discarding observations completely at random. We report complete-data, complete-case, and imputation analyses, by using stratified and within-study imputation methods. We used stratified, fixed-effects, and random-effects meta-analysis methods. In the inverse-variance weighted analyses, results are given where Rubin's rules and meta-analysis have been applied in both orders. For each imputation model, we generated 50 imputed datasets.

Table III shows the results. There was considerable heterogeneity between the studies, with *I*^2^ = 77*%* (95% CI: 58%, 87%) in the complete-data meta-analysis. The only analysis where the imputation brings the point estimate closer to the complete-data estimate and reduces the standard error of the estimate (but not to be lower than that from the complete-data analysis) is the random-effects meta-analysis using the within-study imputation model and using Rubin's rules then meta-analyzing. This was the preferred method from the simulation study when there is between-study heterogeneity, where the efficiency of the multiple imputation analysis is close to that of the complete-data analysis. Concerningly, using the stratified imputation model and the random-effects analysis model, the precision of the multiple imputation analysis is greater than that of the complete-data analysis, and using the stratified analysis model, precision of the multiple imputation analyses is less than that of the complete-case analysis. Table SA7 shows further details of the data in this example.

**Table III:** Regression coefficients for the association of low-density lipoprotein cholesterol (mmol/L) with systolic blood pressure (mmHg) adjusting for body mass index (kg/m^2^) from complete-data, complete-case, and multiple imputation analyses with stratified and within-study imputation models using stratified, fixed-effects, and random-effects meta-analysis models: estimates with standard error (in brackets). In inverse-variance weighted analyses, it is indicated whether Rubin's rules were applied within each study prior to meta-analysis (RR then MA) or meta-analysis of imputed datasets was performed prior to combining estimates using Rubin's rules (MA then RR).

Analysis model:		Stratified	Fixed-effect	Random-effects
Complete-data		1.219 (0.078)	1.084 (0.069)	1.189 (0.225)
Complete-case		1.230 (0.088)	1.105 (0.078)	1.166 (0.231)
Stratified imputation	(MA then RR)	1.248 (0.093)	1.093 (0.081)	1.278 (0.220)
	(RR then MA)		1.099 (0.077)	1.278 (0.211)
Within-study imputation	(MA then RR)	1.236 (0.089)	1.112 (0.079)	1.165 (0.239)
	(RR then MA)		1.110 (0.078)	1.177 (0.226)

## 5. Discussion

In this paper, we have considered combining multiple imputation and meta-analysis using simulated and real data. Two main issues have been addressed: the order for applying Rubin's rules and an inverse-variance weighted meta-analysis, and the congeniality of the imputation and analysis models.

In our simulation study, imputing missing data from a model that allows for between-study heterogeneity induced heterogeneity between studies in a meta-analysis even where there was no heterogeneity in the original data. This resulted in poor coverage properties in a fixed-effect meta-analysis model whichever order of Rubin's rules and the meta-analysis of studies was applied, even when there was no heterogeneity in the data-generating mechanism for the parameter of interest. A random-effects meta-analysis of the study-specific estimates combined by Rubin's rules (Rubin's rules then meta-analysis) gave pooled estimates with the correct coverage level; we overestimated confidence intervals when Rubin's rules were applied to pooled estimates from imputed datasets (meta-analysis then Rubin's rules).

### 5.1. Congeniality of the imputation and analysis models

Use of congenial imputation and analysis models has a more fundamental impact on meta-analysis results. We considered a stratified imputation model, where the same coefficients for each covariate and the same error distribution were assumed across studies, and a within-study imputation model, where the coefficients and error distributions were estimated separately for each study. The stratified imputation model is congenial to the stratified analysis model, as both models can be derived from the same underlying joint model. Similarly, the within-study imputation model (with Rubin's rules applied at the study level) is congenial to both the fixed-effects and the random-effects inverse-variance weighted analysis models. Otherwise, the imputation and analysis models are not congenial. For example, for a stratified imputation model with an inverse-variance weighted analysis model, the imputer assumes more than the analyst. In this case, standard errors for the parameter of interest will generally be too small, and coverage may be below the nominal level.

In our simulations, the stratified imputation model performed moderately well for the stratified analysis method, but poorly for the inverse-variance weighted analysis methods. The within-study imputation method performed well for the inverse-variance weighted analysis methods, especially under a random-effects model, but showed some bias and reduced coverage with the stratified analysis method, although the bias was less than for the inverse-variance weighted methods with the stratified imputation model. Although the stratified analysis method performs well in this paper in the absence of heterogeneity in the parameter of interest, we caution over its use in practice, as it is likely that additional between-study variability beyond that considered in this paper may be present.

In their paper, Robins and Wang [25] gave examples showing that, when the imputer assumes more than the analyst: (i) if the imputation and/or analysis model are misspecified, bias in the standard error can be in either direction; and (ii) if the imputation and analysis models are correctly specified, bias in the standard error is upward. In contrast to this, we found a downward bias in the standard error in the case of Scenario 1 using a stratified imputation model and a fixed-effect analysis model where the imputation and analysis models are correctly specified, but the imputation model additionally (correctly) assumes homogeneity of variance parameters, although the standard error in the random-effects analysis was seemingly unbiased. This appears to be a specific property of the meta-analysis context: Imputation across studies using a stratified model induced correlation between the imputed data values, and between the parameter estimates from each study, leading to overly precise estimates in the fixed-effect analysis model.

### 5.2. Imputation of systematically missing covariates

Two methods have been proposed for the imputation of systematically missing covariates, that is, covariates, which are completely missing in a study. The first method [24] relies on imputation with a fixed error distribution for all studies similar to the stratified imputation model considered here. This method suffers from bias, which may be due to the uncongeniality of the imputation and analysis models as demonstrated in this paper. However, this method is for a multilevel (one-stage) analysis model, which is not considered in this paper, which focuses on inverse-variance weighted (two-stage) meta-analyses. The second method [26] relies on a multivariate meta-analysis of the regression coefficients for the variable of interest under different models of covariate adjustment. In a simple case where studies measure a set of covariates either *U*_1_ or (*U*_1_,*U*_2_) and a fully adjusted estimate is required, we take inference from the bivariate meta-analysis of regression coefficients for the model of outcome on the variable adjusting for *U*_1_ and coefficients for the model adjusting for (*U*_1_,*U*_2_). A disadvantage of this method is the additional complexity if studies measure multiple different combinations of covariates, and the lack of generalizability to missing data on the outcome or variable of interest.

One possibility for imputing data on a completely missing covariate without making restrictive assumptions about its variance is by using information on measured covariates in that study to estimate the variance of the systematically missing covariate. We could consider a multivariate meta-analysis model for the standard deviation of all covariates. This would mean that studies with large variances of the measured covariates would have large estimated values for the variance of the missing covariate. We could implement this in a Bayesian framework, and we would acknowledge uncertainty in the estimate of the variance of the covariate throughout the model. We would require further detailed simulations to establish the validity and efficiency of such a method compared with established methods.

### 5.3. Fixed-effects and random-effects on covariates

The two imputation models considered in this paper can be thought of as extreme cases with respect to the pooling of coefficients and error distributions across studies. We could use a homoscedastic model for the imputation (or analysis) model, with different coefficients in each study but the same error distribution across studies. We can perform this by allowing random-effects distributions on the regression coefficients in a hierarchical model [27]. We could implement such a model in a likelihood framework using mlwin [28] or a Bayesian framework [29]. Theoretically, we could use a random-effects distribution for the variance in each study, although it is not clear what distribution would be appropriate (a normal distribution on the log-transformed standard deviation parameters could be used), or how to implement such a method in practice.

### 5.4. Survival data

Although the context of this paper has been for the imputation of continuous covariates with a continuous outcome, the main outcome of interest in the ERFC dataset is a survival outcome. We would need further simulations to establish the behavior of the methods considered for survival data.

### 5.5. Conclusion

In conclusion, with sporadically missing data in a meta-analysis, congeniality of the imputation and analysis models is important for obtaining valid estimates. In a random-effects meta-analysis, we should apply Rubin's rules at the study level prior to the pooling of study-specific estimates, and we should use a within-study imputation model. The scope of these conclusions is limited by the simulation nature of the analysis and the limitation to considering a linear analysis model with only two covariates, but we have no reason to believe that the findings would not also apply more generally. We need further work to address the issue of how to achieve congeniality of imputation and analysis models with systematically missing covariates.

## References

[1] Rubin D (1976). Inference and missing data. Biometrika.

[2] Little R, Rubin D (2002). Statistical Analysis with Missing Data.

[3] Schafer J (1999). Multiple imputation: a primer. Statistical Methods in Medical Research.

[4] Rubin D (1987). Multiple Imputation for Nonresponse in Surveys.

[5] White I, Carlin J (2010). Bias and efficiency of multiple imputation compared with complete-case analysis for missing covariate values. Statistics in Medicine.

[6] Riley R, Lambert P, Abo-Zaid G (2010). Meta-analysis of individual participant data: rationale, conduct, and reporting. British Medical Journal.

[7] Simmonds M, Higgins J, Stewart L, Tierney J, Clarke M, Thompson S (2005). Meta-analysis of individual patient data from randomized trials: a review of methods used in practice. Clinical Trials.

[8] Thompson S, Sharp S (1999). Explaining heterogeneity in meta-analysis: a comparison of methods. Statistics in Medicine.

[9] Stewart L, Parmar M (1993). Meta-analysis of the literature or of individual patient data: is there a difference?. The Lancet.

[10] Schafer J, Yucel R (2002). Computational strategies for multivariate linear mixed-effects models with missing values. Journal of Computational and Graphical Statistics.

[11] van Buuren S (2011). The Handbook of Advanced Multilevel Analysis.

[12] Koopman L, van der Heijden G, Grobbee D, Rovers M (2008). Comparison of methods of handling missing data in individual patient data meta-analyses: an empirical example on antibiotics in children with acute otitis media. American Journal of Epidemiology.

[13] Meng X (1994). Multiple-imputation inferences with uncongenial sources of input. Statistical Science.

[14] Emerging Risk Factors Collaboration (2007). The Emerging Risk Factors Collaboration: analysis of individual data on lipid, inflammatory and other markers in over 1.1 million participants in 104 prospective studies of cardiovascular diseases. European Journal of Epidemiology.

[15] DerSimonian R, Laird N (1986). Meta-analysis in clinical trials. Controlled Clinical Trials.

[16] Borenstein M, Hedges L, Higgins J, Rothstein H (2009). Introduction to Meta-Analysis. Chapter 34: Generality of the Basic Inverse-Variance Method.

[17] White I (2009). Multivariate random-effects meta-analysis. Stata Journal.

[18] Arnold B, Castillo E, Sarabia J (2001). Conditionally specified distributions: an introduction (with comments and a rejoinder by the authors). Statistical Science.

[19] StataCorp (2011).

[20] R Development Core Team (2011). R: A Language and Environment for Statistical Computing.

[21] Higgins J, Thompson S, Spiegelhalter D (2009). A re-evaluation of random-effects meta-analysis. Journal of the Royal Statistical Society: Series A (Statistics in Society).

[22] Schafer J (1997). Analysis of Incomplete Multivariate Data.

[23] Andridge R (2011). Quantifying the impact of fixed effects modeling of clusters in multiple imputation for cluster randomized trials. Biometrical Journal.

[24] Resche-Rigon M, White I, Barlett J, Thompson S

[25] Robins J, Wang N (2000). Inference for imputation estimators. Biometrika.

[26] Fibrinogen Studies Collaboration (2009). Systematically missing confounders in individual participant data meta-analysis of observational cohort studies. Statistics in Medicine.

[27] Yucel R (2008). Multiple imputation inference for multivariate multilevel continuous data with ignorable non-response. Philosophical Transactions of the Royal Society A: Mathematical, Physical and Engineering Sciences.

[28] Rasbash J, Steele F, Browne W, Goldstein H (2009).

[29] Spiegelhalter D, Thomas A, Best N, Lunn D (2003).

