# Supplementary material for: Combining multiple imputation and meta-analysis with individual participant data
Source: Stat Med. 2013 May 24;32(26):4499–514. doi: 10.1002/sim.5844 (PMC3963448; doi:10.1002/sim.5844)
Supplement: Supplementary file 1 [file sim0032-4499-sd1.pdf]

# Web Appendix

## A.1 Results from simulation study with unequal sized studies

Results are given from the simulation study with unequal sized studies in Web Tables A1 and A2.

## A.2 Results from simulation study with five studies

In response to a reviewer's concerns, we repeated the simulation study using the same parameters except with five equal sized results. Results are given in Web Tables A3 and A4. Estimates in these and the next tables with five and ten studies are more prone to random variation in the mean, standard deviation and mean standard error of estimates due to the smaller number of participants. The Monte Carlo standard error for the mean estimate in each case can be calculated by dividing the standard deviation of estimates by the square root of 1000. The Monte Carlo standard error for the coverage is fixed in all instances at 0.7%.

## A.3 Results from simulation study with ten studies

We also repeated the simulation study using the same parameters except with ten equal sized results. Results are given in Web Tables A5 and A6.

## A.4 Details of the applied analysis

Details of the ten studies included in the applied example of Section 4 are given in Web Table A7. We note that there is considerable variation between the studies in each of the parameters considered in the simulated example of the paper.

| Missing data approach | Stratified analysis Mean | SD | SE | Cov % | Fixed-effect analysis Mean | SD | SE | Cov % | Random-effects analysis Mean | SD | SE | Cov % |
|---|---|---|---|---|---|---|---|---|---|---|---|---|
| **Scenario 1** | | | | | | | | | | | | |
| Complete-data | 0.300 | 0.013 | 0.013 | 95.0 | 0.300 | 0.013 | 0.013 | 94.5 | 0.300 | 0.013 | 0.014 | 95.4 |
| Complete-case | 0.300 | 0.021 | 0.021 | 94.4 | 0.300 | 0.021 | 0.020 | 94.5 | 0.300 | 0.021 | 0.022 | 95.9 |
| Stratified (MA then RR) | 0.301 | 0.016 | 0.016 | 94.0 | 0.301 | 0.016 | 0.016 | 93.9 | 0.301 | 0.016 | 0.016 | 94.7 |
| Stratified (RR then MA) | | | | | 0.300 | 0.016 | 0.014 | 92.1 | 0.300 | 0.016 | 0.014 | 92.1 |
| Within-study (MA then RR) | 0.298 | 0.016 | 0.016 | 94.3 | 0.299 | 0.016 | 0.016 | 94.7 | 0.299 | 0.016 | 0.020 | 98.6 |
| Within-study (RR then MA) | | | | | 0.298 | 0.016 | 0.016 | 93.9 | 0.298 | 0.016 | 0.017 | 95.5 |
| **Scenario 2** | | | | | | | | | | | | |
| Complete-data | 0.300 | 0.014 | 0.014 | 95.0 | 0.300 | 0.011 | 0.012 | 94.8 | 0.300 | 0.011 | 0.012 | 96.2 |
| Complete-case | 0.300 | 0.021 | 0.021 | 95.7 | 0.300 | 0.018 | 0.018 | 94.5 | 0.300 | 0.018 | 0.019 | 95.3 |
| Stratified (MA then RR) | 0.300 | 0.016 | 0.016 | 95.8 | 0.291 | 0.015 | 0.015 | 91.8 | 0.293 | 0.015 | 0.016 | 94.6 |
| Stratified (RR then MA) | | | | | 0.292 | 0.015 | 0.014 | 89.6 | 0.292 | 0.015 | 0.014 | 89.6 |
| Within-study (MA then RR) | 0.297 | 0.016 | 0.016 | 95.2 | 0.298 | 0.015 | 0.015 | 94.9 | 0.298 | 0.015 | 0.019 | 98.8 |
| Within-study (RR then MA) | | | | | 0.298 | 0.015 | 0.014 | 94.1 | 0.298 | 0.015 | 0.015 | 95.8 |
| **Scenario 3** | | | | | | | | | | | | |
| Complete-data | 0.299 | 0.013 | 0.014 | 94.4 | 0.300 | 0.012 | 0.012 | 94.1 | 0.300 | 0.012 | 0.013 | 95.0 |
| Complete-case | 0.298 | 0.021 | 0.021 | 96.0 | 0.299 | 0.018 | 0.018 | 94.9 | 0.299 | 0.018 | 0.020 | 96.4 |
| Stratified (MA then RR) | 0.300 | 0.017 | 0.016 | 94.2 | 0.291 | 0.017 | 0.015 | 87.8 | 0.294 | 0.017 | 0.020 | 96.7 |
| Stratified (RR then MA) | | | | | 0.293 | 0.017 | 0.014 | 92.2 | 0.294 | 0.017 | 0.017 | 92.2 |
| Within-study (MA then RR) | 0.296 | 0.016 | 0.016 | 93.2 | 0.298 | 0.016 | 0.015 | 93.0 | 0.298 | 0.016 | 0.019 | 97.7 |
| Within-study (RR then MA) | | | | | 0.297 | 0.016 | 0.015 | 91.8 | 0.297 | 0.016 | 0.016 | 93.6 |
| **Scenario 4** | | | | | | | | | | | | |
| Complete-data | 0.299 | 0.016 | 0.014 | 92.6 | 0.300 | 0.012 | 0.012 | 94.1 | 0.300 | 0.012 | 0.013 | 95.5 |
| Complete-case | 0.299 | 0.024 | 0.022 | 93.0 | 0.300 | 0.019 | 0.018 | 94.0 | 0.300 | 0.019 | 0.020 | 95.5 |
| Stratified (MA then RR) | 0.300 | 0.019 | 0.017 | 92.7 | 0.293 | 0.017 | 0.015 | 89.2 | 0.295 | 0.017 | 0.020 | 96.1 |
| Stratified (RR then MA) | | | | | 0.294 | 0.017 | 0.014 | 93.5 | 0.296 | 0.017 | 0.017 | 93.5 |
| Within-study (MA then RR) | 0.297 | 0.019 | 0.017 | 91.9 | 0.299 | 0.016 | 0.015 | 93.3 | 0.299 | 0.016 | 0.019 | 97.8 |
| Within-study (RR then MA) | | | | | 0.298 | 0.016 | 0.014 | 93.3 | 0.298 | 0.016 | 0.016 | 95.1 |
| **Scenario 5** | | | | | | | | | | | | |
| Complete-data | 0.301 | 0.025 | 0.014 | 71.1 | 0.301 | 0.026 | 0.012 | 62.3 | 0.301 | 0.023 | 0.023 | 94.0 |
| Complete-case | 0.301 | 0.031 | 0.022 | 83.7 | 0.301 | 0.030 | 0.018 | 77.6 | 0.301 | 0.028 | 0.027 | 93.5 |
| Stratified (MA then RR) | 0.302 | 0.027 | 0.017 | 77.0 | 0.295 | 0.028 | 0.015 | 72.9 | 0.298 | 0.026 | 0.026 | 93.6 |
| Stratified (RR then MA) | | | | | 0.296 | 0.027 | 0.014 | 91.8 | 0.298 | 0.026 | 0.024 | 91.8 |
| Within-study (MA then RR) | 0.298 | 0.027 | 0.017 | 76.2 | 0.299 | 0.028 | 0.015 | 70.9 | 0.300 | 0.025 | 0.027 | 95.4 |
| Within-study (RR then MA) | | | | | 0.299 | 0.028 | 0.014 | 69.1 | 0.300 | 0.025 | 0.024 | 93.5 |

Web Table A1: Simulation study comparing complete-data, complete-case and multiple imputation analyses with stratified and within-study imputation models to estimate $\beta_1 = 0.3$ with thirty (30) unequal sized studies using stratified, and fixed- and random-effects meta-analysis models in five scenarios with increasing heterogeneity: mean estimate across simulations, standard deviation (SD) of estimates, mean standard error (SE) estimate, and coverage (Cov %) of the 95% confidence interval. In inverse-variance weighted analyses, it is indicated whether Rubin's rules were applied within each study prior to meta-analysis (RR then MA) or meta-analysis of imputed datasets was performed prior to combining estimates using Rubin's rules (MA then RR).

| Missing data approach | Stratified analysis Mean | SD | SE | Cov % | Fixed-effect analysis Mean | SD | SE | Cov % | Random-effects analysis Mean | SD | SE | Cov % |
|---|---|---|---|---|---|---|---|---|---|---|---|---|
| Scenario 1 | | | | | | | | | | | | |
| Complete-data | -0.600 | 0.013 | 0.013 | 95.5 | -0.600 | 0.013 | 0.013 | 95.3 | -0.600 | 0.013 | 0.014 | 96.1 |
| Complete-case | -0.600 | 0.019 | 0.019 | 94.8 | -0.600 | 0.019 | 0.019 | 94.5 | -0.600 | 0.019 | 0.020 | 95.7 |
| Stratified (MA then RR) | -0.600 | 0.017 | 0.017 | 94.4 | -0.600 | 0.017 | 0.017 | 94.8 | -0.600 | 0.017 | 0.018 | 95.5 |
| Stratified (RR then MA) | | | | | -0.599 | 0.017 | 0.015 | 91.6 | -0.599 | 0.017 | 0.015 | 91.6 |
| Within-study (MA then RR) | -0.584 | 0.018 | 0.018 | 83.6 | -0.597 | 0.018 | 0.017 | 92.5 | -0.595 | 0.018 | 0.023 | 98.1 |
| Within-study (RR then MA) | | | | | -0.600 | 0.018 | 0.017 | 93.2 | -0.599 | 0.018 | 0.018 | 95.4 |
| Scenario 2 | | | | | | | | | | | | |
| Complete-data | -0.601 | 0.014 | 0.014 | 95.0 | -0.600 | 0.012 | 0.012 | 93.5 | -0.600 | 0.012 | 0.012 | 95.1 |
| Complete-case | -0.601 | 0.019 | 0.020 | 96.0 | -0.601 | 0.017 | 0.016 | 94.1 | -0.601 | 0.017 | 0.018 | 95.3 |
| Stratified (MA then RR) | -0.600 | 0.019 | 0.018 | 93.8 | -0.562 | 0.020 | 0.017 | 40.5 | -0.582 | 0.020 | 0.024 | 91.5 |
| Stratified (RR then MA) | | | | | -0.566 | 0.020 | 0.015 | 40.2 | -0.578 | 0.020 | 0.020 | 75.7 |
| Within-study (MA then RR) | -0.586 | 0.019 | 0.019 | 87.2 | -0.597 | 0.016 | 0.015 | 92.5 | -0.596 | 0.017 | 0.022 | 98.6 |
| Within-study (RR then MA) | | | | | -0.599 | 0.016 | 0.015 | 91.9 | -0.599 | 0.016 | 0.016 | 93.5 |
| Scenario 3 | | | | | | | | | | | | |
| Complete-data | -0.600 | 0.013 | 0.014 | 95.0 | -0.600 | 0.012 | 0.012 | 95.9 | -0.600 | 0.012 | 0.013 | 96.7 |
| Complete-case | -0.600 | 0.020 | 0.020 | 94.3 | -0.600 | 0.017 | 0.017 | 95.0 | -0.600 | 0.017 | 0.018 | 96.3 |
| Stratified (MA then RR) | -0.601 | 0.019 | 0.018 | 93.5 | -0.556 | 0.019 | 0.017 | 28.3 | -0.576 | 0.019 | 0.024 | 87.8 |
| Stratified (RR then MA) | | | | | -0.560 | 0.019 | 0.015 | 27.3 | -0.572 | 0.020 | 0.020 | 69.8 |
| Within-study (MA then RR) | -0.586 | 0.018 | 0.018 | 86.3 | -0.597 | 0.015 | 0.015 | 93.5 | -0.596 | 0.017 | 0.022 | 98.3 |
| Within-study (RR then MA) | | | | | -0.599 | 0.016 | 0.015 | 92.9 | -0.599 | 0.016 | 0.017 | 95.2 |
| Scenario 4 | | | | | | | | | | | | |
| Complete-data | -0.599 | 0.041 | 0.014 | 49.2 | -0.597 | 0.047 | 0.012 | 40.7 | -0.599 | 0.040 | 0.039 | 93.3 |
| Complete-case | -0.598 | 0.044 | 0.020 | 63.6 | -0.596 | 0.049 | 0.017 | 51.0 | -0.598 | 0.043 | 0.041 | 92.7 |
| Stratified (MA then RR) | -0.600 | 0.043 | 0.019 | 60.6 | -0.540 | 0.046 | 0.018 | 27.5 | -0.579 | 0.042 | 0.035 | 84.5 |
| Stratified (RR then MA) | | | | | -0.541 | 0.046 | 0.015 | 23.4 | -0.576 | 0.042 | 0.032 | 79.5 |
| Within-study (MA then RR) | -0.584 | 0.043 | 0.019 | 58.8 | -0.594 | 0.048 | 0.016 | 49.6 | -0.593 | 0.042 | 0.043 | 94.2 |
| Within-study (RR then MA) | | | | | -0.603 | 0.048 | 0.015 | 45.4 | -0.597 | 0.042 | 0.040 | 92.7 |
| Scenario 5 | | | | | | | | | | | | |
| Complete-data | -0.599 | 0.040 | 0.014 | 51.1 | -0.600 | 0.047 | 0.012 | 36.7 | -0.599 | 0.039 | 0.039 | 93.6 |
| Complete-case | -0.600 | 0.043 | 0.020 | 63.8 | -0.600 | 0.050 | 0.017 | 47.8 | -0.600 | 0.042 | 0.041 | 93.7 |
| Stratified (MA then RR) | -0.601 | 0.043 | 0.019 | 60.5 | -0.539 | 0.046 | 0.018 | 27.7 | -0.578 | 0.041 | 0.035 | 85.6 |
| Stratified (RR then MA) | | | | | -0.541 | 0.046 | 0.015 | 22.6 | -0.575 | 0.042 | 0.032 | 79.7 |
| Within-study (MA then RR) | -0.585 | 0.042 | 0.019 | 58.2 | -0.597 | 0.049 | 0.016 | 47.1 | -0.594 | 0.042 | 0.043 | 94.7 |
| Within-study (RR then MA) | | | | | -0.606 | 0.049 | 0.015 | 43.2 | -0.597 | 0.041 | 0.040 | 93.6 |

Web Table A2: Simulation study comparing complete-data, complete-case and multiple imputation analyses with stratified and within-study imputation models to estimate $\beta_2 = -0.6$ with thirty (30) unequal sized studies using stratified, and fixed- and random-effects meta-analysis models in five scenarios with increasing heterogeneity: mean estimate across simulations, standard deviation (SD) of estimates, mean standard error (SE) estimate, and coverage (Cov %) of the 95% confidence interval. In inverse-variance weighted analyses, it is indicated whether Rubin's rules were applied within each study prior to meta-analysis (RR then MA) or meta-analysis of imputed datasets was performed prior to combining estimates using Rubin's rules (MA then RR).

| Missing data approach | Stratified analysis Mean | SD | SE | Cov % | Fixed-effect analysis Mean | SD | SE | Cov % | Random-effects analysis Mean | SD | SE | Cov % |
|---|---|---|---|---|---|---|---|---|---|---|---|---|
| Scenario 1 | | | | | | | | | | | | |
| Complete-data | 0.300 | 0.033 | 0.032 | 94.8 | 0.300 | 0.033 | 0.032 | 94.5 | 0.300 | 0.033 | 0.036 | 95.8 |
| Complete-case | 0.301 | 0.051 | 0.050 | 94.8 | 0.302 | 0.051 | 0.050 | 94.8 | 0.301 | 0.051 | 0.056 | 95.9 |
| Stratified (MA then RR) | 0.299 | 0.039 | 0.039 | 93.5 | 0.300 | 0.039 | 0.039 | 93.5 | 0.300 | 0.039 | 0.042 | 95.3 |
| Stratified (RR then MA) | | | | | 0.299 | 0.039 | 0.036 | 92.5 | 0.299 | 0.039 | 0.038 | 93.1 |
| Within-study (MA then RR) | 0.297 | 0.040 | 0.039 | 93.9 | 0.298 | 0.040 | 0.039 | 93.4 | 0.298 | 0.040 | 0.049 | 97.4 |
| Within-study (RR then MA) | | | | | 0.297 | 0.040 | 0.038 | 93.7 | 0.297 | 0.040 | 0.044 | 95.7 |
| Scenario 2 | | | | | | | | | | | | |
| Complete-data | 0.301 | 0.032 | 0.033 | 95.9 | 0.301 | 0.028 | 0.029 | 95.3 | 0.301 | 0.029 | 0.032 | 96.6 |
| Complete-case | 0.303 | 0.052 | 0.052 | 95.5 | 0.302 | 0.046 | 0.044 | 93.7 | 0.303 | 0.046 | 0.051 | 95.6 |
| Stratified (MA then RR) | 0.302 | 0.039 | 0.040 | 94.9 | 0.295 | 0.035 | 0.037 | 95.6 | 0.297 | 0.036 | 0.041 | 96.6 |
| Stratified (RR then MA) | | | | | 0.296 | 0.036 | 0.034 | 94.3 | 0.297 | 0.036 | 0.036 | 95.3 |
| Within-study (MA then RR) | 0.301 | 0.038 | 0.040 | 95.8 | 0.301 | 0.036 | 0.036 | 94.0 | 0.302 | 0.036 | 0.046 | 98.5 |
| Within-study (RR then MA) | | | | | 0.301 | 0.036 | 0.035 | 94.9 | 0.301 | 0.036 | 0.040 | 96.7 |
| Scenario 3 | | | | | | | | | | | | |
| Complete-data | 0.301 | 0.034 | 0.033 | 95.1 | 0.301 | 0.030 | 0.029 | 95.0 | 0.301 | 0.031 | 0.033 | 96.4 |
| Complete-case | 0.300 | 0.052 | 0.052 | 94.2 | 0.300 | 0.048 | 0.045 | 93.6 | 0.300 | 0.049 | 0.051 | 95.6 |
| Stratified (MA then RR) | 0.301 | 0.042 | 0.040 | 94.0 | 0.293 | 0.042 | 0.037 | 90.4 | 0.296 | 0.041 | 0.048 | 95.9 |
| Stratified (RR then MA) | | | | | 0.295 | 0.042 | 0.034 | 88.7 | 0.296 | 0.042 | 0.043 | 93.9 |
| Within-study (MA then RR) | 0.299 | 0.041 | 0.040 | 94.4 | 0.299 | 0.038 | 0.036 | 93.1 | 0.300 | 0.040 | 0.047 | 97.2 |
| Within-study (RR then MA) | | | | | 0.299 | 0.038 | 0.036 | 93.6 | 0.299 | 0.039 | 0.041 | 94.8 |
| Scenario 4 | | | | | | | | | | | | |
| Complete-data | 0.300 | 0.038 | 0.034 | 92.5 | 0.300 | 0.030 | 0.029 | 95.1 | 0.300 | 0.031 | 0.033 | 97.0 |
| Complete-case | 0.300 | 0.057 | 0.053 | 92.5 | 0.301 | 0.047 | 0.045 | 93.5 | 0.301 | 0.049 | 0.052 | 95.0 |
| Stratified (MA then RR) | 0.299 | 0.046 | 0.041 | 91.7 | 0.293 | 0.042 | 0.037 | 90.6 | 0.295 | 0.042 | 0.048 | 96.0 |
| Stratified (RR then MA) | | | | | 0.295 | 0.042 | 0.035 | 89.5 | 0.296 | 0.042 | 0.043 | 93.8 |
| Within-study (MA then RR) | 0.297 | 0.044 | 0.040 | 92.8 | 0.299 | 0.038 | 0.036 | 93.0 | 0.298 | 0.039 | 0.048 | 97.2 |
| Within-study (RR then MA) | | | | | 0.298 | 0.038 | 0.036 | 93.4 | 0.298 | 0.038 | 0.042 | 95.6 |
| Scenario 5 | | | | | | | | | | | | |
| Complete-data | 0.300 | 0.094 | 0.035 | 54.5 | 0.301 | 0.108 | 0.029 | 41.7 | 0.300 | 0.093 | 0.090 | 88.3 |
| Complete-case | 0.300 | 0.105 | 0.054 | 67.5 | 0.301 | 0.115 | 0.045 | 56.4 | 0.300 | 0.102 | 0.098 | 88.3 |
| Stratified (MA then RR) | 0.300 | 0.098 | 0.041 | 59.3 | 0.294 | 0.103 | 0.038 | 52.9 | 0.296 | 0.096 | 0.088 | 87.3 |
| Stratified (RR then MA) | | | | | 0.295 | 0.103 | 0.035 | 49.7 | 0.296 | 0.096 | 0.084 | 85.1 |
| Within-study (MA then RR) | 0.297 | 0.097 | 0.041 | 58.2 | 0.299 | 0.111 | 0.038 | 51.0 | 0.299 | 0.096 | 0.098 | 91.0 |
| Within-study (RR then MA) | | | | | 0.298 | 0.108 | 0.036 | 50.4 | 0.299 | 0.096 | 0.093 | 89.2 |

Web Table A3: Simulation study comparing complete-data, complete-case and multiple imputation analyses with stratified and within-study imputation models to estimate $\beta_1 = 0.3$ with five (5) equal sized studies using stratified, and fixed- and random-effects meta-analysis models in five scenarios with increasing heterogeneity: mean estimate across simulations, standard deviation (SD) of estimates, mean standard error (SE) estimate, and coverage (Cov %) of the 95% confidence interval. In inverse-variance weighted analyses, it is indicated whether Rubin's rules were applied within each study prior to meta-analysis (RR then MA) or meta-analysis of imputed datasets was performed prior to combining estimates using Rubin's rules (MA then RR).

| Missing data approach | Stratified analysis Mean | SD | SE | Cov % | Fixed-effect analysis Mean | SD | SE | Cov % | Random-effects analysis Mean | SD | SE | Cov % |
|---|---|---|---|---|---|---|---|---|---|---|---|---|
| Scenario 1 | | | | | | | | | | | | |
| Complete-data | -0.600 | 0.032 | 0.032 | 94.9 | -0.599 | 0.033 | 0.032 | 94.7 | -0.599 | 0.032 | 0.036 | 96.0 |
| Complete-case | -0.599 | 0.045 | 0.046 | 95.5 | -0.599 | 0.046 | 0.046 | 96.0 | -0.599 | 0.045 | 0.051 | 96.4 |
| Stratified (MA then RR) | -0.597 | 0.042 | 0.042 | 94.1 | -0.597 | 0.043 | 0.042 | 94.0 | -0.597 | 0.042 | 0.045 | 95.3 |
| Stratified (RR then MA) | | | | | -0.596 | 0.043 | 0.038 | 91.4 | -0.597 | 0.043 | 0.039 | 92.0 |
| Within-study (MA then RR) | -0.584 | 0.042 | 0.043 | 93.4 | -0.595 | 0.043 | 0.043 | 93.9 | -0.593 | 0.043 | 0.057 | 98.6 |
| Within-study (RR then MA) | | | | | -0.598 | 0.043 | 0.041 | 93.8 | -0.596 | 0.043 | 0.047 | 95.9 |
| Scenario 2 | | | | | | | | | | | | |
| Complete-data | -0.600 | 0.033 | 0.033 | 94.6 | -0.600 | 0.030 | 0.029 | 93.5 | -0.600 | 0.030 | 0.032 | 95.0 |
| Complete-case | -0.599 | 0.048 | 0.047 | 95.1 | -0.599 | 0.042 | 0.040 | 94.7 | -0.599 | 0.043 | 0.046 | 95.9 |
| Stratified (MA then RR) | -0.598 | 0.046 | 0.044 | 93.6 | -0.566 | 0.047 | 0.041 | 82.1 | -0.583 | 0.046 | 0.057 | 96.1 |
| Stratified (RR then MA) | | | | | -0.570 | 0.048 | 0.037 | 79.9 | -0.580 | 0.047 | 0.048 | 91.0 |
| Within-study (MA then RR) | -0.587 | 0.045 | 0.045 | 93.9 | -0.597 | 0.039 | 0.037 | 93.4 | -0.596 | 0.042 | 0.054 | 97.7 |
| Within-study (RR then MA) | | | | | -0.599 | 0.040 | 0.037 | 93.5 | -0.599 | 0.041 | 0.043 | 95.0 |
| Scenario 3 | | | | | | | | | | | | |
| Complete-data | -0.599 | 0.032 | 0.033 | 95.4 | -0.599 | 0.029 | 0.029 | 95.2 | -0.599 | 0.029 | 0.033 | 96.3 |
| Complete-case | -0.600 | 0.048 | 0.047 | 94.4 | -0.599 | 0.043 | 0.041 | 94.1 | -0.599 | 0.043 | 0.047 | 95.9 |
| Stratified (MA then RR) | -0.599 | 0.046 | 0.044 | 94.3 | -0.561 | 0.047 | 0.042 | 79.4 | -0.579 | 0.046 | 0.059 | 95.1 |
| Stratified (RR then MA) | | | | | -0.565 | 0.047 | 0.038 | 77.4 | -0.577 | 0.047 | 0.050 | 87.4 |
| Within-study (MA then RR) | -0.587 | 0.045 | 0.045 | 94.1 | -0.597 | 0.039 | 0.038 | 94.8 | -0.595 | 0.042 | 0.055 | 98.9 |
| Within-study (RR then MA) | | | | | -0.599 | 0.041 | 0.037 | 93.0 | -0.598 | 0.041 | 0.044 | 95.7 |
| Scenario 4 | | | | | | | | | | | | |
| Complete-data | -0.605 | 0.097 | 0.034 | 51.1 | -0.607 | 0.109 | 0.029 | 39.5 | -0.605 | 0.097 | 0.090 | 87.5 |
| Complete-case | -0.604 | 0.102 | 0.049 | 64.6 | -0.605 | 0.113 | 0.041 | 53.1 | -0.604 | 0.101 | 0.095 | 87.0 |
| Stratified (MA then RR) | -0.604 | 0.100 | 0.046 | 62.8 | -0.557 | 0.106 | 0.043 | 52.6 | -0.587 | 0.098 | 0.083 | 85.8 |
| Stratified (RR then MA) | | | | | -0.558 | 0.108 | 0.038 | 49.5 | -0.584 | 0.099 | 0.075 | 80.1 |
| Within-study (MA then RR) | -0.592 | 0.099 | 0.046 | 62.5 | -0.603 | 0.111 | 0.039 | 53.1 | -0.599 | 0.100 | 0.102 | 91.4 |
| Within-study (RR then MA) | | | | | -0.609 | 0.112 | 0.037 | 50.7 | -0.602 | 0.100 | 0.093 | 87.2 |
| Scenario 5 | | | | | | | | | | | | |
| Complete-data | -0.601 | 0.101 | 0.035 | 52.0 | -0.602 | 0.111 | 0.029 | 41.0 | -0.601 | 0.098 | 0.088 | 86.1 |
| Complete-case | -0.601 | 0.104 | 0.049 | 64.5 | -0.602 | 0.115 | 0.041 | 50.4 | -0.601 | 0.101 | 0.094 | 87.8 |
| Stratified (MA then RR) | -0.603 | 0.104 | 0.046 | 59.8 | -0.547 | 0.107 | 0.043 | 51.8 | -0.578 | 0.100 | 0.082 | 83.7 |
| Stratified (RR then MA) | | | | | -0.548 | 0.107 | 0.038 | 48.3 | -0.575 | 0.101 | 0.074 | 78.8 |
| Within-study (MA then RR) | -0.587 | 0.103 | 0.047 | 61.6 | -0.597 | 0.114 | 0.040 | 50.1 | -0.595 | 0.101 | 0.101 | 92.0 |
| Within-study (RR then MA) | | | | | -0.605 | 0.115 | 0.038 | 48.2 | -0.598 | 0.101 | 0.092 | 87.5 |

Web Table A4: Simulation study comparing complete-data, complete-case and multiple imputation analyses with stratified and within-study imputation models to estimate $\beta_2 = -0.6$ with five (5) equal sized studies using stratified, and fixed- and random-effects meta-analysis models in five scenarios with increasing heterogeneity: mean estimate across simulations, standard deviation (SD) of estimates, mean standard error (SE) estimate, and coverage (Cov %) of the 95% confidence interval. In inverse-variance weighted analyses, it is indicated whether Rubin's rules were applied within each study prior to meta-analysis (RR then MA) or meta-analysis of imputed datasets was performed prior to combining estimates using Rubin's rules (MA then RR).

| | Stratified analysis | | | | Fixed-effect analysis | | | | Random-effects analysis | | | |
|---|---|---|---|---|---|---|---|---|---|---|---|---|
| Missing data approach | Mean | SD | SE | Cov % | Mean | SD | SE | Cov % | Mean | SD | SE | Cov % |
| Scenario 1 | | | | | | | | | | | | |
| Complete-data | 0.300 | 0.023 | 0.023 | 95.2 | 0.300 | 0.023 | 0.023 | 95.2 | 0.300 | 0.023 | 0.025 | 96.1 |
| Complete-case | 0.300 | 0.035 | 0.036 | 95.7 | 0.300 | 0.036 | 0.035 | 95.0 | 0.300 | 0.036 | 0.039 | 96.5 |
| Stratified (MA then RR) | 0.299 | 0.027 | 0.027 | 94.7 | 0.299 | 0.027 | 0.027 | 94.6 | 0.299 | 0.027 | 0.029 | 95.6 |
| Stratified (RR then MA) | | | | | 0.299 | 0.027 | 0.025 | 93.3 | 0.299 | 0.027 | 0.026 | 94.1 |
| Within-study (MA then RR) | 0.296 | 0.027 | 0.028 | 94.5 | 0.297 | 0.028 | 0.028 | 94.6 | 0.297 | 0.028 | 0.034 | 98.2 |
| Within-study (RR then MA) | | | | | 0.296 | 0.028 | 0.027 | 94.2 | 0.297 | 0.028 | 0.030 | 96.0 |
| Scenario 2 | | | | | | | | | | | | |
| Complete-data | 0.300 | 0.024 | 0.024 | 95.1 | 0.301 | 0.021 | 0.020 | 94.2 | 0.300 | 0.021 | 0.022 | 95.7 |
| Complete-case | 0.300 | 0.038 | 0.037 | 93.5 | 0.301 | 0.033 | 0.030 | 92.9 | 0.301 | 0.033 | 0.034 | 94.8 |
| Stratified (MA then RR) | 0.300 | 0.029 | 0.028 | 93.9 | 0.292 | 0.026 | 0.026 | 93.5 | 0.293 | 0.026 | 0.028 | 95.3 |
| Stratified (RR then MA) | | | | | 0.292 | 0.026 | 0.024 | 91.3 | 0.293 | 0.026 | 0.025 | 91.8 |
| Within-study (MA then RR) | 0.297 | 0.029 | 0.028 | 94.6 | 0.299 | 0.026 | 0.025 | 93.3 | 0.299 | 0.027 | 0.032 | 97.5 |
| Within-study (RR then MA) | | | | | 0.298 | 0.026 | 0.024 | 92.5 | 0.298 | 0.027 | 0.027 | 94.3 |
| Scenario 3 | | | | | | | | | | | | |
| Complete-data | 0.300 | 0.024 | 0.024 | 95.3 | 0.301 | 0.020 | 0.020 | 94.2 | 0.301 | 0.021 | 0.022 | 96.0 |
| Complete-case | 0.299 | 0.037 | 0.037 | 95.2 | 0.300 | 0.033 | 0.031 | 94.3 | 0.300 | 0.033 | 0.034 | 95.8 |
| Stratified (MA then RR) | 0.299 | 0.029 | 0.028 | 94.8 | 0.291 | 0.030 | 0.026 | 90.1 | 0.294 | 0.028 | 0.034 | 97.6 |
| Stratified (RR then MA) | | | | | 0.293 | 0.030 | 0.024 | 87.7 | 0.294 | 0.029 | 0.029 | 93.8 |
| Within-study (MA then RR) | 0.297 | 0.028 | 0.028 | 94.7 | 0.298 | 0.026 | 0.026 | 94.7 | 0.298 | 0.027 | 0.033 | 98.1 |
| Within-study (RR then MA) | | | | | 0.298 | 0.026 | 0.025 | 93.6 | 0.298 | 0.027 | 0.028 | 95.7 |
| Scenario 4 | | | | | | | | | | | | |
| Complete-data | 0.299 | 0.027 | 0.024 | 91.4 | 0.298 | 0.021 | 0.020 | 94.3 | 0.298 | 0.021 | 0.022 | 96.0 |
| Complete-case | 0.298 | 0.040 | 0.038 | 94.1 | 0.298 | 0.033 | 0.031 | 93.0 | 0.298 | 0.034 | 0.035 | 94.4 |
| Stratified (MA then RR) | 0.298 | 0.032 | 0.029 | 92.0 | 0.290 | 0.030 | 0.026 | 90.0 | 0.293 | 0.030 | 0.034 | 96.4 |
| Stratified (RR then MA) | | | | | 0.292 | 0.030 | 0.024 | 88.0 | 0.293 | 0.029 | 0.030 | 93.8 |
| Within-study (MA then RR) | 0.296 | 0.031 | 0.029 | 90.6 | 0.296 | 0.027 | 0.026 | 92.2 | 0.297 | 0.028 | 0.033 | 97.1 |
| Within-study (RR then MA) | | | | | 0.296 | 0.027 | 0.025 | 92.6 | 0.296 | 0.027 | 0.028 | 94.6 |
| Scenario 5 | | | | | | | | | | | | |
| Complete-data | 0.299 | 0.072 | 0.025 | 50.4 | 0.298 | 0.084 | 0.020 | 37.6 | 0.299 | 0.070 | 0.064 | 90.6 |
| Complete-case | 0.299 | 0.079 | 0.038 | 65.7 | 0.297 | 0.088 | 0.031 | 51.3 | 0.298 | 0.076 | 0.069 | 90.3 |
| Stratified (MA then RR) | 0.300 | 0.073 | 0.029 | 56.6 | 0.293 | 0.077 | 0.027 | 52.2 | 0.295 | 0.072 | 0.063 | 89.2 |
| Stratified (RR then MA) | | | | | 0.294 | 0.077 | 0.024 | 48.5 | 0.296 | 0.072 | 0.060 | 87.3 |
| Within-study (MA then RR) | 0.297 | 0.073 | 0.029 | 56.7 | 0.296 | 0.086 | 0.026 | 46.0 | 0.297 | 0.072 | 0.069 | 91.5 |
| Within-study (RR then MA) | | | | | 0.295 | 0.082 | 0.025 | 45.7 | 0.297 | 0.072 | 0.066 | 90.2 |

Web Table A5: Simulation study comparing complete-data, complete-case and multiple imputation analyses with stratified and within-study imputation models to estimate $\beta_1 = 0.3$ with ten (10) equal sized studies using stratified, and fixed- and random-effects meta-analysis models in five scenarios with increasing heterogeneity: mean estimate across simulations, standard deviation (SD) of estimates, mean standard error (SE) estimate, and coverage (Cov %) of the 95% confidence interval. In inverse-variance weighted analyses, it is indicated whether Rubin's rules were applied within each study prior to meta-analysis (RR then MA) or meta-analysis of imputed datasets was performed prior to combining estimates using Rubin's rules (MA then RR).

| | Stratified analysis | | | | Fixed-effect analysis | | | | Random-effects analysis | | | |
|---|---|---|---|---|---|---|---|---|---|---|---|---|
| Missing data approach | Mean | SD | SE | Cov % | Mean | SD | SE | Cov % | Mean | SD | SE | Cov % |
| Scenario 1 | | | | | | | | | | | | |
| Complete-data | -0.600 | 0.023 | 0.023 | 94.6 | -0.600 | 0.023 | 0.023 | 95.0 | -0.600 | 0.023 | 0.025 | 96.0 |
| Complete-case | -0.600 | 0.033 | 0.033 | 94.5 | -0.599 | 0.033 | 0.032 | 94.5 | -0.599 | 0.033 | 0.035 | 96.0 |
| Stratified (MA then RR) | -0.600 | 0.030 | 0.030 | 94.0 | -0.600 | 0.030 | 0.030 | 94.3 | -0.600 | 0.030 | 0.032 | 95.2 |
| Stratified (RR then MA) | | | | | -0.599 | 0.031 | 0.027 | 90.9 | -0.599 | 0.031 | 0.027 | 90.9 |
| Within-study (MA then RR) | -0.585 | 0.030 | 0.030 | 91.4 | -0.597 | 0.030 | 0.030 | 93.3 | -0.595 | 0.031 | 0.040 | 98.4 |
| Within-study (RR then MA) | | | | | -0.600 | 0.031 | 0.029 | 93.1 | -0.599 | 0.031 | 0.032 | 94.9 |
| Scenario 2 | | | | | | | | | | | | |
| Complete-data | -0.600 | 0.024 | 0.024 | 94.8 | -0.600 | 0.020 | 0.020 | 94.4 | -0.600 | 0.020 | 0.022 | 95.8 |
| Complete-case | -0.601 | 0.035 | 0.034 | 95.5 | -0.600 | 0.030 | 0.028 | 94.6 | -0.600 | 0.030 | 0.031 | 96.0 |
| Stratified (MA then RR) | -0.600 | 0.033 | 0.031 | 93.0 | -0.561 | 0.034 | 0.029 | 69.2 | -0.582 | 0.034 | 0.041 | 94.5 |
| Stratified (RR then MA) | | | | | -0.566 | 0.034 | 0.026 | 68.8 | -0.579 | 0.035 | 0.035 | 87.4 |
| Within-study (MA then RR) | -0.586 | 0.033 | 0.032 | 91.7 | -0.597 | 0.027 | 0.026 | 94.3 | -0.595 | 0.029 | 0.037 | 98.5 |
| Within-study (RR then MA) | | | | | -0.599 | 0.027 | 0.025 | 93.3 | -0.598 | 0.028 | 0.028 | 95.2 |
| Scenario 3 | | | | | | | | | | | | |
| Complete-data | -0.601 | 0.023 | 0.024 | 95.6 | -0.600 | 0.020 | 0.020 | 95.7 | -0.600 | 0.020 | 0.022 | 96.4 |
| Complete-case | -0.600 | 0.033 | 0.034 | 95.2 | -0.600 | 0.029 | 0.028 | 94.8 | -0.600 | 0.029 | 0.032 | 97.0 |
| Stratified (MA then RR) | -0.601 | 0.032 | 0.031 | 93.8 | -0.556 | 0.033 | 0.030 | 64.7 | -0.577 | 0.032 | 0.042 | 94.5 |
| Stratified (RR then MA) | | | | | -0.561 | 0.033 | 0.026 | 62.8 | -0.574 | 0.033 | 0.035 | 86.0 |
| Within-study (MA then RR) | -0.587 | 0.032 | 0.032 | 93.2 | -0.597 | 0.027 | 0.026 | 94.0 | -0.596 | 0.029 | 0.038 | 99.0 |
| Within-study (RR then MA) | | | | | -0.599 | 0.028 | 0.026 | 93.8 | -0.598 | 0.028 | 0.029 | 96.1 |
| Scenario 4 | | | | | | | | | | | | |
| Complete-data | -0.600 | 0.069 | 0.024 | 50.0 | -0.599 | 0.084 | 0.020 | 37.5 | -0.600 | 0.069 | 0.065 | 89.9 |
| Complete-case | -0.600 | 0.075 | 0.035 | 62.4 | -0.600 | 0.088 | 0.029 | 48.4 | -0.600 | 0.074 | 0.069 | 90.0 |
| Stratified (MA then RR) | -0.599 | 0.073 | 0.033 | 59.8 | -0.541 | 0.079 | 0.031 | 43.7 | -0.579 | 0.072 | 0.060 | 86.5 |
| Stratified (RR then MA) | | | | | -0.542 | 0.080 | 0.027 | 39.1 | -0.576 | 0.073 | 0.055 | 81.2 |
| Within-study (MA then RR) | -0.586 | 0.073 | 0.033 | 60.1 | -0.596 | 0.086 | 0.028 | 47.2 | -0.594 | 0.073 | 0.073 | 93.7 |
| Within-study (RR then MA) | | | | | -0.603 | 0.086 | 0.026 | 44.9 | -0.597 | 0.073 | 0.067 | 89.8 |
| Scenario 5 | | | | | | | | | | | | |
| Complete-data | -0.605 | 0.069 | 0.025 | 52.0 | -0.602 | 0.082 | 0.020 | 38.6 | -0.604 | 0.067 | 0.066 | 91.2 |
| Complete-case | -0.605 | 0.074 | 0.035 | 65.0 | -0.603 | 0.087 | 0.028 | 49.0 | -0.604 | 0.072 | 0.070 | 91.3 |
| Stratified (MA then RR) | -0.607 | 0.073 | 0.033 | 61.6 | -0.540 | 0.079 | 0.030 | 46.0 | -0.579 | 0.071 | 0.060 | 88.2 |
| Stratified (RR then MA) | | | | | -0.542 | 0.080 | 0.027 | 41.6 | -0.577 | 0.071 | 0.055 | 82.5 |
| Within-study (MA then RR) | -0.591 | 0.073 | 0.033 | 62.3 | -0.600 | 0.084 | 0.027 | 48.9 | -0.599 | 0.071 | 0.074 | 93.2 |
| Within-study (RR then MA) | | | | | -0.607 | 0.086 | 0.026 | 44.6 | -0.602 | 0.072 | 0.068 | 90.4 |

Web Table A6: Simulation study comparing complete-data, complete-case and multiple imputation analyses with stratified and within-study imputation models to estimate $\beta_2 = -0.6$ with ten (10) equal sized studies using stratified, and fixed- and random-effects meta-analysis models in five scenarios with increasing heterogeneity: mean estimate across simulations, standard deviation (SD) of estimates, mean standard error (SE) estimate, and coverage (Cov %) of the 95% confidence interval. In inverse-variance weighted analyses, it is indicated whether Rubin's rules were applied within each study prior to meta-analysis (RR then MA) or meta-analysis of imputed datasets was performed prior to combining estimates using Rubin's rules (MA then RR).

| Study | $N$ | Variables: mean (SD) LDL-C | BMI | SBP | LDL-C/BMI correlation | Association of LDL-C on SBP Complete-data | Complete-case |
|---|---|---|---|---|---|---|---|
| GOTOW | 5785 | 3.74 (0.86) | 26.23 (2.99) | 132.0 (15.9) | 0.12 | 1.22 (0.24) | 1.18 (0.26) |
| KAREL72 | 2059 | 4.01 (0.99) | 26.75 (3.50) | 130.7 (17.2) | -0.01 | 0.91 (0.37) | 0.85 (0.40) |
| MGERAUG1 | 3963 | 3.91 (1.13) | 27.04 (4.22) | 134.0 (19.3) | 0.17 | 2.40 (0.26) | 2.46 (0.29) |
| MGERAUG2 | 3373 | 3.80 (1.08) | 27.67 (4.40) | 135.8 (19.7) | 0.15 | 1.78 (0.31) | 1.89 (0.35) |
| NHANES3 | 694 | 3.51 (0.94) | 25.89 (5.06) | 129.1 (14.5) | 0.12 | 1.06 (0.57) | 0.59 (0.65) |
| PROCAM | 3253 | 3.78 (0.81) | 26.94 (4.37) | 156.7 (21.5) | 0.04 | 0.06 (0.46) | -0.02 (0.52) |
| WHIHABPS | 1576 | 1.55 (0.43) | 27.25 (5.43) | 133.0 (19.2) | 0.16 | 3.78 (1.15) | 3.37 (1.30) |
| WHITE1 | 3874 | 3.38 (0.78) | 25.18 (3.23) | 145.3 (19.9) | 0.08 | 0.64 (0.41) | 0.47 (0.45) |
| WHS | 22932 | 3.23 (0.89) | 26.36 (5.07) | 127.0 (11.9) | 0.09 | 0.95 (0.08) | 0.98 (0.10) |
| WOSCOPS | 6214 | 4.95 (0.45) | 25.92 (3.16) | 135.4 (17.3) | 0.01 | 0.42 (0.48) | 0.78 (0.54) |

Web Table A7: Summary of studies: number of participants ($N$), mean and standard deviation (SD) of low density lipoprotein-cholesterol (LDL-C), body mass index (BMI), and systolic blood pressure (SBP), correlation between LDL-C and BMI, coefficients of association of LDL-C on SBP adjusting for BMI with standard errors from complete-data and complete-case analyses

## A.5 Alternative tables of results

For the main simulation studies presented in the paper with 30 studies, in order to more directly compare the performance of imputation methods in each scenario, alternative tables are presented displaying the same results, but grouped by imputation method rather than by scenario.

| | Stratified analysis | | | | Fixed-effect analysis | | | | Random-effects analysis | | | |
|---|---|---|---|---|---|---|---|---|---|---|---|---|
| | Mean | SD | SE | Coverage | Mean | SD | SE | Coverage | Mean | SD | SE | Coverage |
| Complete-data analysis | | | | | | | | | | | | |
| Scenario 1 | 0.300 | 0.013 | 0.013 | 95.1 | 0.300 | 0.013 | 0.013 | 94.9 | 0.300 | 0.013 | 0.014 | 95.7 |
| Scenario 2 | 0.300 | 0.013 | 0.014 | 96.2 | 0.300 | 0.011 | 0.012 | 96.0 | 0.300 | 0.011 | 0.012 | 97.1 |
| Scenario 3 | 0.300 | 0.014 | 0.014 | 95.7 | 0.300 | 0.012 | 0.012 | 94.4 | 0.300 | 0.012 | 0.013 | 95.5 |
| Scenario 4 | 0.301 | 0.016 | 0.014 | 90.9 | 0.300 | 0.012 | 0.012 | 94.5 | 0.300 | 0.012 | 0.013 | 95.9 |
| Scenario 5 | 0.299 | 0.024 | 0.014 | 76.0 | 0.299 | 0.025 | 0.012 | 65.5 | 0.299 | 0.023 | 0.023 | 95.1 |
| Complete-case analysis | | | | | | | | | | | | |
| Scenario 1 | 0.301 | 0.021 | 0.021 | 93.8 | 0.300 | 0.021 | 0.020 | 93.6 | 0.300 | 0.021 | 0.022 | 95.0 |
| Scenario 2 | 0.301 | 0.022 | 0.021 | 94.8 | 0.300 | 0.019 | 0.018 | 93.6 | 0.300 | 0.019 | 0.019 | 94.7 |
| Scenario 3 | 0.299 | 0.021 | 0.021 | 94.9 | 0.299 | 0.019 | 0.018 | 94.6 | 0.299 | 0.019 | 0.020 | 95.8 |
| Scenario 4 | 0.301 | 0.023 | 0.022 | 93.7 | 0.300 | 0.019 | 0.018 | 93.8 | 0.300 | 0.019 | 0.020 | 94.9 |
| Scenario 5 | 0.299 | 0.029 | 0.022 | 85.6 | 0.299 | 0.029 | 0.018 | 77.7 | 0.299 | 0.027 | 0.027 | 94.1 |
| Stratified imputation method (MA then RR) | | | | | | | | | | | | |
| Scenario 1 | 0.300 | 0.016 | 0.016 | 93.4 | 0.300 | 0.016 | 0.016 | 93.2 | 0.300 | 0.016 | 0.016 | 94.3 |
| Scenario 2 | 0.300 | 0.016 | 0.016 | 95.5 | 0.291 | 0.014 | 0.015 | 91.9 | 0.292 | 0.015 | 0.016 | 94.2 |
| Scenario 3 | 0.300 | 0.016 | 0.016 | 94.8 | 0.291 | 0.016 | 0.015 | 87.8 | 0.293 | 0.016 | 0.020 | 97.0 |
| Scenario 4 | 0.301 | 0.018 | 0.017 | 91.9 | 0.293 | 0.017 | 0.015 | 88.1 | 0.295 | 0.017 | 0.020 | 97.1 |
| Scenario 5 | 0.299 | 0.025 | 0.017 | 78.9 | 0.292 | 0.026 | 0.015 | 73.0 | 0.295 | 0.024 | 0.026 | 95.4 |
| Stratified imputation method (RR then MA) | | | | | | | | | | | | |
| Scenario 1 | | | | | 0.300 | 0.016 | 0.014 | 91.1 | 0.300 | 0.016 | 0.014 | 91.4 |
| Scenario 2 | | | | | 0.292 | 0.014 | 0.014 | 89.6 | 0.292 | 0.014 | 0.014 | 90.0 |
| Scenario 3 | | | | | 0.292 | 0.017 | 0.014 | 85.9 | 0.293 | 0.016 | 0.017 | 94.0 |
| Scenario 4 | | | | | 0.294 | 0.017 | 0.014 | 86.9 | 0.296 | 0.017 | 0.017 | 94.2 |
| Scenario 5 | | | | | 0.294 | 0.026 | 0.014 | 68.8 | 0.295 | 0.024 | 0.024 | 93.0 |
| Within-study imputation method (MA then RR) | | | | | | | | | | | | |
| Scenario 1 | 0.297 | 0.016 | 0.016 | 93.2 | 0.298 | 0.016 | 0.016 | 92.0 | 0.298 | 0.016 | 0.020 | 97.7 |
| Scenario 2 | 0.297 | 0.016 | 0.016 | 95.2 | 0.298 | 0.015 | 0.015 | 93.7 | 0.298 | 0.015 | 0.019 | 98.6 |
| Scenario 3 | 0.297 | 0.016 | 0.016 | 95.4 | 0.298 | 0.015 | 0.015 | 94.1 | 0.298 | 0.015 | 0.019 | 98.1 |
| Scenario 4 | 0.298 | 0.018 | 0.016 | 92.3 | 0.299 | 0.015 | 0.015 | 94.0 | 0.299 | 0.015 | 0.019 | 98.1 |
| Scenario 5 | 0.296 | 0.025 | 0.017 | 79.7 | 0.298 | 0.026 | 0.015 | 72.8 | 0.297 | 0.024 | 0.027 | 96.2 |
| Within-study imputation method (RR then MA) | | | | | | | | | | | | |
| Scenario 1 | | | | | 0.297 | 0.017 | 0.016 | 91.9 | 0.297 | 0.017 | 0.017 | 93.1 |
| Scenario 2 | | | | | 0.297 | 0.015 | 0.014 | 93.6 | 0.298 | 0.015 | 0.015 | 94.9 |
| Scenario 3 | | | | | 0.297 | 0.015 | 0.015 | 93.1 | 0.297 | 0.015 | 0.016 | 94.7 |
| Scenario 4 | | | | | 0.298 | 0.015 | 0.014 | 93.7 | 0.298 | 0.015 | 0.016 | 95.2 |
| Scenario 5 | | | | | 0.298 | 0.026 | 0.014 | 72.2 | 0.297 | 0.024 | 0.024 | 94.3 |

Web Table A8: Simulation study comparing complete-data, complete-case and multiple imputation analyses with stratified and within-study imputation models to estimate $\beta_1 = 0.3$ with thirty (30) equal sized studies using stratified, and fixed- and random-effects meta-analysis models in five scenarios with increasing heterogeneity: mean estimate across simulations, standard deviation (SD) of estimates, mean standard error (SE) estimate, and coverage (%) of the 95% confidence interval. In inverse-variance weighted analyses, it is indicated whether Rubin's rules were applied within each study prior to meta-analysis (RR then MA) or meta-analysis of imputed datasets was performed prior to combining estimates using Rubin's rules (MA then RR).

| | Stratified analysis | | | | Fixed-effect analysis | | | | Random-effects analysis | | | |
|---|---|---|---|---|---|---|---|---|---|---|---|---|
| | Mean | SD | SE | Coverage | Mean | SD | SE | Coverage | Mean | SD | SE | Coverage |
| Complete-data analysis | | | | | | | | | | | | |
| Scenario 1 | -0.600 | 0.013 | 0.013 | 95.0 | -0.600 | 0.013 | 0.013 | 95.0 | -0.600 | 0.013 | 0.014 | 95.8 |
| Scenario 2 | -0.600 | 0.014 | 0.014 | 95.1 | -0.600 | 0.012 | 0.012 | 94.3 | -0.600 | 0.012 | 0.012 | 95.5 |
| Scenario 3 | -0.599 | 0.014 | 0.014 | 94.6 | -0.600 | 0.012 | 0.012 | 95.0 | -0.600 | 0.012 | 0.013 | 95.8 |
| Scenario 4 | -0.602 | 0.040 | 0.014 | 50.2 | -0.602 | 0.045 | 0.012 | 38.0 | -0.602 | 0.040 | 0.039 | 94.8 |
| Scenario 5 | -0.598 | 0.039 | 0.014 | 53.1 | -0.599 | 0.045 | 0.012 | 39.4 | -0.598 | 0.038 | 0.039 | 93.4 |
| Complete-case analysis | | | | | | | | | | | | |
| Scenario 1 | -0.600 | 0.019 | 0.019 | 93.9 | -0.600 | 0.020 | 0.019 | 93.3 | -0.600 | 0.020 | 0.020 | 95.1 |
| Scenario 2 | -0.600 | 0.020 | 0.020 | 94.2 | -0.600 | 0.017 | 0.016 | 93.8 | -0.600 | 0.017 | 0.018 | 94.7 |
| Scenario 3 | -0.599 | 0.020 | 0.020 | 94.5 | -0.599 | 0.017 | 0.017 | 94.4 | -0.599 | 0.017 | 0.018 | 95.2 |
| Scenario 4 | -0.602 | 0.042 | 0.020 | 64.1 | -0.602 | 0.047 | 0.017 | 51.5 | -0.602 | 0.041 | 0.041 | 94.4 |
| Scenario 5 | -0.599 | 0.041 | 0.020 | 66.1 | -0.600 | 0.048 | 0.017 | 50.0 | -0.599 | 0.041 | 0.041 | 94.5 |
| Stratified imputation method (MA then RR) | | | | | | | | | | | | |
| Scenario 1 | -0.600 | 0.018 | 0.017 | 93.2 | -0.600 | 0.018 | 0.017 | 93.2 | -0.600 | 0.018 | 0.018 | 93.9 |
| Scenario 2 | -0.600 | 0.019 | 0.018 | 92.2 | -0.562 | 0.019 | 0.017 | 40.0 | -0.581 | 0.019 | 0.024 | 90.3 |
| Scenario 3 | -0.600 | 0.019 | 0.018 | 92.4 | -0.554 | 0.019 | 0.017 | 27.7 | -0.575 | 0.020 | 0.024 | 85.1 |
| Scenario 4 | -0.603 | 0.042 | 0.019 | 62.1 | -0.543 | 0.045 | 0.018 | 29.9 | -0.582 | 0.041 | 0.035 | 86.3 |
| Scenario 5 | -0.600 | 0.040 | 0.019 | 63.3 | -0.540 | 0.043 | 0.018 | 27.2 | -0.577 | 0.039 | 0.035 | 84.7 |
| Stratified imputation method (RR then MA) | | | | | | | | | | | | |
| Scenario 1 | | | | | -0.599 | 0.018 | 0.015 | 90.3 | -0.599 | 0.018 | 0.015 | 90.4 |
| Scenario 2 | | | | | -0.566 | 0.019 | 0.015 | 38.5 | -0.577 | 0.020 | 0.019 | 76.5 |
| Scenario 3 | | | | | -0.558 | 0.020 | 0.015 | 28.9 | -0.570 | 0.020 | 0.020 | 67.3 |
| Scenario 4 | | | | | -0.545 | 0.045 | 0.015 | 26.8 | -0.579 | 0.041 | 0.032 | 81.9 |
| Scenario 5 | | | | | -0.541 | 0.043 | 0.015 | 22.9 | -0.575 | 0.040 | 0.032 | 79.7 |
| Within-study imputation method (MA then RR) | | | | | | | | | | | | |
| Scenario 1 | -0.585 | 0.018 | 0.018 | 85.1 | -0.597 | 0.018 | 0.017 | 92.2 | -0.595 | 0.018 | 0.023 | 97.8 |
| Scenario 2 | -0.585 | 0.019 | 0.019 | 86.3 | -0.597 | 0.016 | 0.015 | 92.8 | -0.595 | 0.017 | 0.022 | 98.4 |
| Scenario 3 | -0.585 | 0.019 | 0.018 | 85.1 | -0.597 | 0.016 | 0.015 | 94.1 | -0.595 | 0.017 | 0.022 | 98.6 |
| Scenario 4 | -0.587 | 0.040 | 0.019 | 60.5 | -0.599 | 0.046 | 0.016 | 49.7 | -0.596 | 0.041 | 0.043 | 95.5 |
| Scenario 5 | -0.584 | 0.040 | 0.019 | 59.6 | -0.597 | 0.046 | 0.016 | 49.4 | -0.593 | 0.040 | 0.043 | 96.3 |
| Within-study imputation method (RR then MA) | | | | | | | | | | | | |
| Scenario 1 | | | | | -0.600 | 0.019 | 0.017 | 92.0 | -0.599 | 0.019 | 0.018 | 93.5 |
| Scenario 2 | | | | | -0.599 | 0.016 | 0.015 | 92.0 | -0.598 | 0.016 | 0.016 | 94.4 |
| Scenario 3 | | | | | -0.599 | 0.016 | 0.015 | 93.2 | -0.598 | 0.016 | 0.017 | 95.5 |
| Scenario 4 | | | | | -0.608 | 0.047 | 0.015 | 47.6 | -0.599 | 0.041 | 0.040 | 93.6 |
| Scenario 5 | | | | | -0.604 | 0.047 | 0.015 | 45.3 | -0.596 | 0.040 | 0.040 | 94.6 |

Web Table A9: Simulation study comparing complete-data, complete-case and multiple imputation analyses with stratified and within-study imputation models to estimate $\beta_2 = -0.6$ with thirty (30) equal sized studies using stratified, and fixed- and random-effects meta-analysis models in five scenarios with increasing heterogeneity: mean estimate across simulations, standard deviation (SD) of estimates, mean standard error (SE) estimate, and coverage (%) of the 95% confidence interval. In inverse-variance weighted analyses, it is indicated whether Rubin's rules were applied within each study prior to meta-analysis (RR then MA) or meta-analysis of imputed datasets was performed prior to combining estimates using Rubin's rules (MA then RR).

| | Stratified analysis | | | | Fixed-effect analysis | | | | Random-effects analysis | | | |
|---|---|---|---|---|---|---|---|---|---|---|---|---|
| | Mean | SD | SE | Coverage | Mean | SD | SE | Coverage | Mean | SD | SE | Coverage |
| Complete-data analysis | | | | | | | | | | | | |
| Scenario 1 | 0.300 | 0.013 | 0.013 | 95.0 | 0.300 | 0.013 | 0.013 | 94.5 | 0.300 | 0.013 | 0.014 | 95.4 |
| Scenario 2 | 0.300 | 0.014 | 0.014 | 95.0 | 0.300 | 0.011 | 0.012 | 94.8 | 0.300 | 0.011 | 0.012 | 96.2 |
| Scenario 3 | 0.299 | 0.013 | 0.014 | 94.4 | 0.300 | 0.012 | 0.012 | 94.1 | 0.300 | 0.012 | 0.013 | 95.0 |
| Scenario 4 | 0.299 | 0.016 | 0.014 | 92.6 | 0.300 | 0.012 | 0.012 | 94.1 | 0.300 | 0.012 | 0.013 | 95.5 |
| Scenario 5 | 0.301 | 0.025 | 0.014 | 71.1 | 0.301 | 0.026 | 0.012 | 62.3 | 0.301 | 0.023 | 0.023 | 94.0 |
| Complete-case analysis | | | | | | | | | | | | |
| Scenario 1 | 0.300 | 0.021 | 0.021 | 94.4 | 0.300 | 0.021 | 0.020 | 94.5 | 0.300 | 0.021 | 0.022 | 95.9 |
| Scenario 2 | 0.300 | 0.021 | 0.021 | 95.7 | 0.300 | 0.018 | 0.018 | 94.5 | 0.300 | 0.018 | 0.019 | 95.3 |
| Scenario 3 | 0.298 | 0.021 | 0.021 | 96.0 | 0.299 | 0.018 | 0.018 | 94.9 | 0.299 | 0.018 | 0.020 | 96.4 |
| Scenario 4 | 0.299 | 0.024 | 0.022 | 93.0 | 0.300 | 0.019 | 0.018 | 94.0 | 0.300 | 0.019 | 0.020 | 95.5 |
| Scenario 5 | 0.301 | 0.031 | 0.022 | 83.7 | 0.301 | 0.030 | 0.018 | 77.6 | 0.301 | 0.028 | 0.027 | 93.5 |
| Stratified imputation method (MA then RR) | | | | | | | | | | | | |
| Scenario 1 | 0.301 | 0.016 | 0.016 | 94.0 | 0.301 | 0.016 | 0.016 | 93.9 | 0.301 | 0.016 | 0.016 | 94.7 |
| Scenario 2 | 0.300 | 0.016 | 0.016 | 95.8 | 0.291 | 0.015 | 0.015 | 91.8 | 0.293 | 0.015 | 0.016 | 94.6 |
| Scenario 3 | 0.300 | 0.017 | 0.016 | 94.2 | 0.291 | 0.017 | 0.015 | 87.8 | 0.294 | 0.017 | 0.020 | 96.7 |
| Scenario 4 | 0.300 | 0.019 | 0.017 | 92.7 | 0.293 | 0.017 | 0.015 | 89.2 | 0.295 | 0.017 | 0.020 | 96.1 |
| Scenario 5 | 0.302 | 0.027 | 0.017 | 77.0 | 0.295 | 0.028 | 0.015 | 72.9 | 0.298 | 0.026 | 0.026 | 93.6 |
| Stratified imputation method (RR then MA) | | | | | | | | | | | | |
| Scenario 1 | | | | | 0.300 | 0.016 | 0.014 | 92.1 | 0.300 | 0.016 | 0.014 | 92.1 |
| Scenario 2 | | | | | 0.292 | 0.015 | 0.014 | 89.6 | 0.292 | 0.015 | 0.014 | 89.6 |
| Scenario 3 | | | | | 0.293 | 0.017 | 0.014 | 92.2 | 0.294 | 0.017 | 0.017 | 92.2 |
| Scenario 4 | | | | | 0.294 | 0.017 | 0.014 | 93.5 | 0.296 | 0.017 | 0.017 | 93.5 |
| Scenario 5 | | | | | 0.296 | 0.027 | 0.014 | 91.8 | 0.298 | 0.026 | 0.024 | 91.8 |
| Within-study imputation method (MA then RR) | | | | | | | | | | | | |
| Scenario 1 | 0.298 | 0.016 | 0.016 | 94.3 | 0.299 | 0.016 | 0.016 | 94.7 | 0.299 | 0.016 | 0.020 | 98.6 |
| Scenario 2 | 0.297 | 0.016 | 0.016 | 95.2 | 0.298 | 0.015 | 0.015 | 94.9 | 0.298 | 0.015 | 0.019 | 98.8 |
| Scenario 3 | 0.296 | 0.016 | 0.016 | 93.2 | 0.298 | 0.016 | 0.015 | 93.0 | 0.298 | 0.016 | 0.019 | 97.7 |
| Scenario 4 | 0.297 | 0.019 | 0.017 | 91.9 | 0.299 | 0.016 | 0.015 | 93.3 | 0.299 | 0.016 | 0.019 | 97.8 |
| Scenario 5 | 0.298 | 0.027 | 0.017 | 76.2 | 0.299 | 0.028 | 0.015 | 70.9 | 0.300 | 0.025 | 0.027 | 95.4 |
| Within-study imputation method (RR then MA) | | | | | | | | | | | | |
| Scenario 1 | | | | | 0.298 | 0.016 | 0.016 | 93.9 | 0.298 | 0.016 | 0.017 | 95.5 |
| Scenario 2 | | | | | 0.298 | 0.015 | 0.014 | 94.1 | 0.298 | 0.015 | 0.015 | 95.8 |
| Scenario 3 | | | | | 0.297 | 0.016 | 0.015 | 91.8 | 0.297 | 0.016 | 0.016 | 93.6 |
| Scenario 4 | | | | | 0.298 | 0.016 | 0.014 | 93.3 | 0.298 | 0.016 | 0.016 | 95.1 |
| Scenario 5 | | | | | 0.299 | 0.028 | 0.014 | 69.1 | 0.300 | 0.025 | 0.024 | 93.5 |

Web Table A10: Complete-data, complete-case and multiple imputation analyses with stratified and within-study imputation models of simulation to estimate $\beta_1 = 0.3$ with unequal sized studies using stratified, and fixed- and random-effects meta-analysis models in five scenarios with increasing heterogeneity: mean estimate across simulations, standard deviation (SD) of estimates, mean standard error (SE) estimate, and coverage (%) of the 95% confidence interval. In inverse-variance weighted analyses, it is indicated whether Rubin's rules were applied within each study prior to meta-analysis (RR then MA) or meta-analysis of imputed datasets was performed prior to combining estimates using Rubin's rules (MA then RR).

| | Stratified analysis | | | | Fixed-effect analysis | | | | Random-effects analysis | | | |
|---|---|---|---|---|---|---|---|---|---|---|---|---|
| | Mean | SD | SE | Coverage | Mean | SD | SE | Coverage | Mean | SD | SE | Coverage |
| Complete-data analysis | | | | | | | | | | | | |
| Scenario 1 | -0.600 | 0.013 | 0.013 | 95.5 | -0.600 | 0.013 | 0.013 | 95.3 | -0.600 | 0.013 | 0.014 | 96.1 |
| Scenario 2 | -0.601 | 0.014 | 0.014 | 95.0 | -0.600 | 0.012 | 0.012 | 93.5 | -0.600 | 0.012 | 0.012 | 95.1 |
| Scenario 3 | -0.600 | 0.013 | 0.014 | 95.0 | -0.600 | 0.012 | 0.012 | 95.9 | -0.600 | 0.012 | 0.013 | 96.7 |
| Scenario 4 | -0.599 | 0.041 | 0.014 | 49.2 | -0.597 | 0.047 | 0.012 | 40.7 | -0.599 | 0.040 | 0.039 | 93.3 |
| Scenario 5 | -0.599 | 0.040 | 0.014 | 51.1 | -0.600 | 0.047 | 0.012 | 36.7 | -0.599 | 0.039 | 0.039 | 93.6 |
| Complete-case analysis | | | | | | | | | | | | |
| Scenario 1 | -0.600 | 0.019 | 0.019 | 94.8 | -0.600 | 0.019 | 0.019 | 94.5 | -0.600 | 0.019 | 0.020 | 95.7 |
| Scenario 2 | -0.601 | 0.019 | 0.020 | 96.0 | -0.601 | 0.017 | 0.016 | 94.1 | -0.601 | 0.017 | 0.018 | 95.3 |
| Scenario 3 | -0.600 | 0.020 | 0.020 | 94.3 | -0.600 | 0.017 | 0.017 | 95.0 | -0.600 | 0.017 | 0.018 | 96.3 |
| Scenario 4 | -0.598 | 0.044 | 0.020 | 63.6 | -0.596 | 0.049 | 0.017 | 51.0 | -0.598 | 0.043 | 0.041 | 92.7 |
| Scenario 5 | -0.600 | 0.043 | 0.020 | 63.8 | -0.600 | 0.050 | 0.017 | 47.8 | -0.600 | 0.042 | 0.041 | 93.7 |
| Stratified imputation method (MA then RR) | | | | | | | | | | | | |
| Scenario 1 | -0.600 | 0.017 | 0.017 | 94.4 | -0.600 | 0.017 | 0.017 | 94.8 | -0.600 | 0.017 | 0.018 | 95.5 |
| Scenario 2 | -0.600 | 0.019 | 0.018 | 93.8 | -0.562 | 0.020 | 0.017 | 40.5 | -0.582 | 0.020 | 0.024 | 91.5 |
| Scenario 3 | -0.601 | 0.019 | 0.018 | 93.5 | -0.556 | 0.019 | 0.017 | 28.3 | -0.576 | 0.019 | 0.024 | 87.8 |
| Scenario 4 | -0.600 | 0.043 | 0.019 | 60.6 | -0.540 | 0.046 | 0.018 | 27.5 | -0.579 | 0.042 | 0.035 | 84.5 |
| Scenario 5 | -0.601 | 0.043 | 0.019 | 60.5 | -0.539 | 0.046 | 0.018 | 27.7 | -0.578 | 0.041 | 0.035 | 85.6 |
| Stratified imputation method (RR then MA) | | | | | | | | | | | | |
| Scenario 1 | | | | | -0.599 | 0.017 | 0.015 | 91.6 | -0.599 | 0.017 | 0.015 | 91.6 |
| Scenario 2 | | | | | -0.566 | 0.020 | 0.015 | 40.2 | -0.578 | 0.020 | 0.020 | 75.7 |
| Scenario 3 | | | | | -0.560 | 0.019 | 0.015 | 27.3 | -0.572 | 0.020 | 0.020 | 69.8 |
| Scenario 4 | | | | | -0.541 | 0.046 | 0.015 | 23.4 | -0.576 | 0.042 | 0.032 | 79.5 |
| Scenario 5 | | | | | -0.541 | 0.046 | 0.015 | 22.6 | -0.575 | 0.042 | 0.032 | 79.7 |
| Within-study imputation method (MA then RR) | | | | | | | | | | | | |
| Scenario 1 | -0.584 | 0.018 | 0.018 | 83.6 | -0.597 | 0.018 | 0.017 | 92.5 | -0.595 | 0.018 | 0.023 | 98.1 |
| Scenario 2 | -0.586 | 0.019 | 0.019 | 87.2 | -0.597 | 0.016 | 0.015 | 92.5 | -0.596 | 0.017 | 0.022 | 98.6 |
| Scenario 3 | -0.586 | 0.018 | 0.018 | 86.3 | -0.597 | 0.015 | 0.015 | 93.5 | -0.596 | 0.017 | 0.022 | 98.3 |
| Scenario 4 | -0.584 | 0.043 | 0.019 | 58.8 | -0.594 | 0.048 | 0.016 | 49.6 | -0.593 | 0.042 | 0.043 | 94.2 |
| Scenario 5 | -0.585 | 0.042 | 0.019 | 58.2 | -0.597 | 0.049 | 0.016 | 47.1 | -0.594 | 0.042 | 0.043 | 94.7 |
| Within-study imputation method (RR then MA) | | | | | | | | | | | | |
| Scenario 1 | | | | | -0.600 | 0.018 | 0.017 | 93.2 | -0.599 | 0.018 | 0.018 | 95.4 |
| Scenario 2 | | | | | -0.599 | 0.016 | 0.015 | 91.9 | -0.599 | 0.016 | 0.016 | 93.5 |
| Scenario 3 | | | | | -0.599 | 0.016 | 0.015 | 92.9 | -0.599 | 0.016 | 0.017 | 95.2 |
| Scenario 4 | | | | | -0.603 | 0.048 | 0.015 | 45.4 | -0.597 | 0.042 | 0.040 | 92.7 |
| Scenario 5 | | | | | -0.606 | 0.049 | 0.015 | 43.2 | -0.597 | 0.041 | 0.040 | 93.6 |

Web Table A11: Complete-data, complete-case and multiple imputation analyses with stratified and within-study imputation models of simulation to estimate $\beta_2 = -0.6$ with unequal sized studies using stratified, and fixed- and random-effects meta-analysis models in five scenarios with increasing heterogeneity: mean estimate across simulations, standard deviation (SD) of estimates, mean standard error (SE) estimate, and coverage (%) of the 95% confidence interval. In inverse-variance weighted analyses, it is indicated whether Rubin's rules were applied within each study prior to meta-analysis (RR then MA) or meta-analysis of imputed datasets was performed prior to combining estimates using Rubin's rules (MA then RR).
